# The pigtail macaque (*Macaca nemestrina*) model of COVID-19 reproduces diverse clinical outcomes and reveals new and complex signatures of disease

**DOI:** 10.1371/journal.ppat.1010162

**Published:** 2021-12-20

**Authors:** Alexandra Melton, Lara A. Doyle-Meyers, Robert V. Blair, Cecily Midkiff, Hunter J. Melton, Kasi Russell-Lodrigue, Pyone P. Aye, Faith Schiro, Marissa Fahlberg, Dawn Szeltner, Skye Spencer, Brandon J. Beddingfield, Kelly Goff, Nadia Golden, Toni Penney, Breanna Picou, Krystle Hensley, Kristin E. Chandler, Jessica A. Plante, Kenneth S. Plante, Scott C. Weaver, Chad J. Roy, James A. Hoxie, Hongmei Gao, David C. Montefiori, Joseph L. Mankowski, Rudolf P. Bohm, Jay Rappaport, Nicholas J. Maness

**Affiliations:** 1 Tulane National Primate Research Center, Covington, Louisiana, United States of America; 2 Biomedical Science Training Program, Tulane University School of Medicine, New Orleans, Louisiana, United States of America; 3 Department of Medicine, Tulane University School of Medicine, New Orleans, Louisiana, United States of America; 4 Florida State University, Department of Statistics, Tallahassee, Florida, United States of America; 5 World Reference Center for Emerging Viruses and Arboviruses, Institute for Human Infections and Immunity, University of Texas Medical Branch, Galveston, Texas, United States of America; 6 Department of Microbiology and Immunology, Tulane University School of Medicine, New Orleans, Louisiana, United States of America; 7 Perelman School of Medicine, University of Pennsylvania, Philadelphia, Pennsylvania, United States of America; 8 Duke University Medical Center, Duke Human Vaccine Institute, Durham, North Carolina, United States of America; 9 Department of Molecular and Comparative Pathobiology, Johns Hopkins School of Medicine, Baltimore, Maryland, United States of America; Icahn School of Medicine at Mount Sinai, UNITED STATES

## Abstract

The novel coronavirus SARS-CoV-2, the causative agent of COVID-19 disease, has killed over five million people worldwide as of December 2021 with infections rising again due to the emergence of highly transmissible variants. Animal models that faithfully recapitulate human disease are critical for assessing SARS-CoV-2 viral and immune dynamics, for understanding mechanisms of disease, and for testing vaccines and therapeutics. Pigtail macaques (PTM, *Macaca nemestrina*) demonstrate a rapid and severe disease course when infected with simian immunodeficiency virus (SIV), including the development of severe cardiovascular symptoms that are pertinent to COVID-19 manifestations in humans. We thus proposed this species may likewise exhibit severe COVID-19 disease upon infection with SARS-CoV-2. Here, we extensively studied a cohort of SARS-CoV-2-infected PTM euthanized either 6- or 21-days after respiratory viral challenge. We show that PTM demonstrate largely mild-to-moderate COVID-19 disease. Pulmonary infiltrates were dominated by T cells, including CD4+ T cells that upregulate CD8 and express cytotoxic molecules, as well as virus-targeting T cells that were predominantly CD4+. We also noted increases in inflammatory and coagulation markers in blood, pulmonary pathologic lesions, and the development of neutralizing antibodies. Together, our data demonstrate that SARS-CoV-2 infection of PTM recapitulates important features of COVID-19 and reveals new immune and viral dynamics and thus may serve as a useful animal model for studying pathogenesis and testing vaccines and therapeutics.

## Author summary

The COVID-19 pandemic has claimed the lives of millions in a span of less than two years. Despite the development of several highly effective vaccines, many millions remain unvaccinated, and several highly transmissible variants have emerged, clearly suggesting the need for new approaches to treat those that become severely ill. The development of new drugs will rely on having animal models that reproduce the most severe disease seen in humans. To date, nonhuman primate models have not exhibited this severe disease. In this study we tested whether pigtail macaques (PTM) might exhibit such severe disease, based on previous work showing this species is prone to more rapid and severe disease when infected with other viruses.

## Introduction

In late 2019, a novel coronavirus was found circulating in humans in China. This virus showed substantial genomic similarities with the severe acute respiratory syndrome coronavirus (SARS-CoV) that caused an outbreak and panic in 2003[1] in addition to a number of bat sarbecoviruses[2]; hence, it was named SARS-CoV-2[3]. SARS-CoV-2 is the causative agent of COVID-19 disease and a worldwide pandemic that has killed more than five million persons to date including 790,000 deaths in the United States. Though most infected individuals exhibit no or mild symptoms, a subset experience severe complications, including highly elevated pro-inflammatory cytokines and coagulation biomarkers, acute respiratory distress syndrome (ARDS), and death[4–9]. Most available data suggest that the intensity of the immune response plays a role in determining COVID-19 severity and progression, with severe disease occurring approximately 3-to-4-weeks after initial symptoms[10, 11]. Thus, a deep understanding of the immunopathologic mechanisms of disease in those with advanced disease and of viral clearance in asymptomatic infection and those with mild disease is critical for the development of next generation therapies and vaccines.

Animal models that faithfully recapitulate human disease are needed to assess the roles of particular cell subsets in disease etiology[12, 13]. Various species of nonhuman primates can be infected by SARS-CoV-2 and exhibit disease ranging from mild to severe[14–18]. The use of timed infections with well characterized viral stocks in animals with relatively high genetic similarity with humans allows the dissection of immune responses with nuance and detail not possible in humans. The most widely used species of NHP for COVID-19 research has been the rhesus macaque (*Macaca mulatta*). This model has proved valuable for testing vaccines as viral infection dynamics in this species are robust and well-studied and therefore can be compared between treatment groups[19]. However, SARS-CoV-2-induced disease in this species is generally mild and does not recapitulate the more severe disease seen in a subset of humans[16]. Thus, multiple NHP models are needed to capture the spectrum of disease seen in humans. In this study, we infected pigtail macaques (PTM, *Macaca nemestrina*) with SARS-CoV-2 (WA1/2020 isolate) to assess this novel animal model of COVID-19 disease.

PTM are a unique and valuable animal model for other viral diseases. Simian immunodeficiency virus (SIV) infection of rhesus macaques (RhM) is the most widely used nonhuman primate (NHP) model of HIV/AIDS and is used widely for testing vaccines and cure strategies[20]. However, SIV-associated disease in RhM can take up to several years to develop, somewhat limiting their use for studying disease mechanisms. In contrast, infection of PTM with the same viral isolates leads to rapid disease development with enhanced cardiovascular manifestations relative to RhM, which is of particular relevance to COVID-19 disease[21–24]. Thus, we proposed that SARS-CoV-2 infection of PTM may likewise lead to accelerated COVID-19 disease or demonstrate immune features of disease not detected in other animal models. If so, this species will be valuable for assessing COVID-19 disease mechanisms and for testing novel vaccines and therapeutics. We tracked viral and immune dynamics through the course of infection in a cohort of PTM. We found that disease in this model largely mirrored that observed in RhM but with unique immune features, such as pulmonary infiltration of CD4+ T cells that exhibit antiviral and cytotoxic functions, as is seen in COVID-19 patients[25]. Together, our data characterize, in depth, a novel animal model that may prove useful for assessing moderate COVID-19 disease mechanisms and testing new therapeutics.

## Materials and methods

### Ethics statement

Pigtail macaques used in this study were purpose bred at the University of Washington National Primate Research Center for experiments. Macaques were housed in compliance with the NRC Guide for the Care and Use of Laboratory Animals and the Animal Welfare Act. Animal experiments were approved by the Institutional Animal Care and Use Committee of Tulane University (protocol P0451). The Tulane National Primate Research Center (TNPRC) is fully accredited by AAALAC International, Animal Welfare Assurance No. A3180-01.

During the study, animals were singly housed indoors in climate-controlled conditions with a 12/12-light/dark cycle. All the animals on this study were monitored twice daily to ensure their welfare. Any abnormalities, including those of appetite, stool, and behavior, were recorded and reported to a veterinarian. The animals were fed commercially prepared nonhuman primate diet twice daily. Supplemental foods were provided in the form of fruit, vegetables, and foraging items as part of the TNPRC environmental enrichment program. Water was available ad libitum through an automatic watering system. The TNPRC environmental enrichment program is reviewed and approved by the IACUC semi-annually. Veterinarians in the TNPRC Division of Veterinary Medicine have established procedures to minimize pain and distress using several approaches. Animals were anesthetized with ketamine-HCl (10 mg/kg) or tiletamine/zolazepam (3–8 mg/kg) prior to all procedures. Preemptive and post procedural analgesia (buprenorphine 0.01 mg/kg or buprenorphine sustained-release 0.2 mg/kg SQ) was used for procedures that would likely cause more than momentary pain or distress in humans undergoing the same procedures. The above listed anesthetics and analgesics were used to minimize pain and distress in accordance with the recommendations of the Weatherall Report. The animals were euthanized at the end of the study using methods consistent with recommendations of the American Veterinary Medical Association (AVMA) Panel on euthanasia and per the recommendations of the IACUC. Specifically, the animals were anesthetized with tiletamine/zolazepam (8 mg/kg IM) and given buprenorphine (0.01 mg/kg IM) followed by an overdose of pentobarbital sodium. Death was confirmed using auscultation to confirm the cessation of respiratory and circulatory functions and by the lack of corneal reflexes.

### Animal cohort, viral inoculations, and procedures

**Fig 1:**
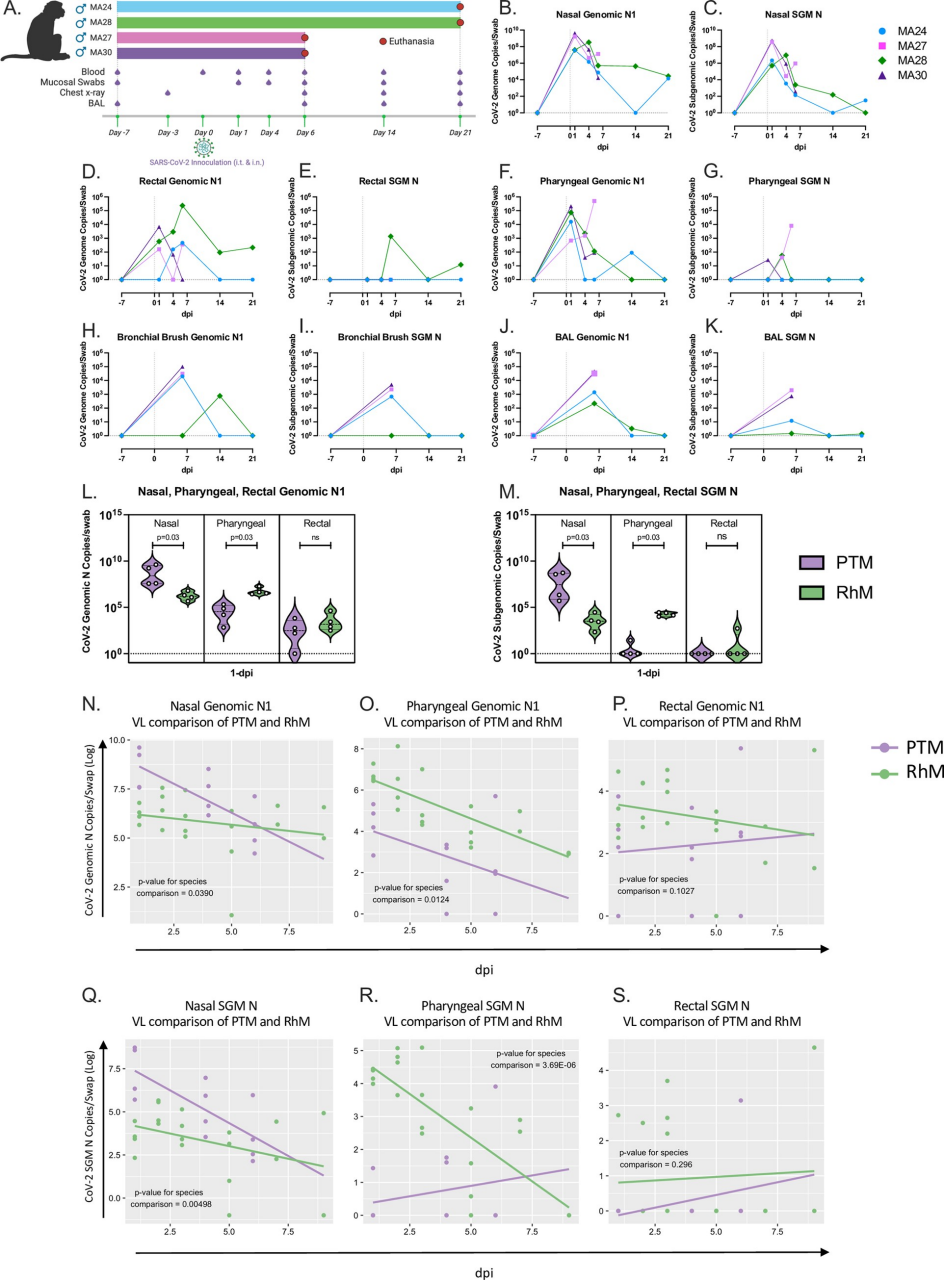
Viral dynamics. A. Outline of study design. Four male pigtail macaques (PTM) were exposed to 1x10^6 TCID50 of SARS-CoV-2 (isolate WA1/2020) through a combination of intranasal (i.n.) and intratracheal (i.t.) inoculation on Day 0. Figure created with BioRender.com. B-K. Quantification of SARS-CoV-2 RNA levels from Pigtail macaque (PTM) mucosal swabs overtime (Quantitative RT PCR). Genomic (B,D,F,H,J) Subgenomic (C,E,G,I,K). L-S. PTM and Rhesus macaque (RhM) viral dynamics (Quantitative RT PCR). Comparison of genomic (L) and subgenomic (SGM, M) SARS-CoV-2 viral titers from nasal, pharyngeal, and rectal mucosal swabs at 1-day post infection (dpi). N-S. Comparison of SARS-CoV-2 RNA levels from nasal (N and Q), pharyngeal (O and R), and rectal (P and S) mucosal swabs overtime. Genomic (N-P) SGM (Q-S). Panels B-K. Baseline: n = 4, Day 1: n = 4, Day 4: n = 4, Day 6: n = 4, Day 14: n = 2, Day 21: n = 2. Panels L-S. Day 1: n = 8, Day 2: n = 4, Day 3: n = 4 Day 4: n = 4, Day 5: n = 4, Day 6: n = 4, Day 7: n = 2, Day 9: n = 2. Mann-Whitney comparison of means (Panels L and M) or a linear regression t-test (Panels N-S) was used to determine significance.

Four male pigtail macaques (PTM), between the ages of 5 and 6 years old (Table 1, Fig 1A and S1 Table), were exposed to 1x10^6^ TCID_50_ of SARS-CoV-2 USA WA1/2020 (World Reference Center for Emerging Viruses and Arboviruses, Galveston, TX) through both intranasal and intratracheal inoculation. The viral stock was sequenced and determined to have no mutations at greater than 5% of reads that differed from the original patient isolate. Pre- and post-exposure samples included blood, bronchoalveolar lavage (BAL), and mucosal swabs (nasal, pharyngeal, rectal, and bronchial brush). Physical examination and imaging (radiography S1 Fig) were conducted before viral exposure and weekly after exposure. Animals were monitored for 6 (n = 2) or 21 (n = 2) days before euthanasia and tissue harvest. At necropsy, samples from each of the major lung lobes (left and right, cranial, middle, and caudal lobes) were collected in TRIzol (Invitrogen, Lithuania) and fresh frozen at -80°C. The remainder of the lung lobes were infused and then immersed in formalin fixative. The rest of the necropsy was performed routinely with collection of tissues from all major organs in DMEM media, fresh frozen, or in formalin fixative.

**Table 1:** PTM cohort used in this study, including sex and age and weight at the time of SARS-CoV-2 inoculation.

Animal ID	Sex	Age (y)	Weight (kg)
MA27	Male	6.07	7.55
MA30	Male	5.61	5.80
MA24	Male	5.64	8.40
MA28	Male	5.81	8.60

### Isolation of Viral RNA

The *Quick*-RNA Viral Kit (Zymo Research, Irvine, CA) was used to isolate viral RNA (vRNA) from mucosal swab and bronchial brush samples collected in 200 μL DNA/RNA Shield 1X (Zymo Research, Irvine, CA) following the manufacturer’s protocol. Briefly, 400 μL DNA/RNA viral buffer was added to the swab samples. In a modification to the manufacturer’s protocol, swabs were transferred directly to the Zymo spin column for centrifugation. The vRNA was eluted in 50 μL elution buffer.

### Viral RNA Quantification by Quantitative Real-Time PCR

Quantification of viral RNA was performed as described[26] using the CDC N1 primers/probe for quantification of total viral RNA and with primers/probe specific for the nucleocapsid subgenomic RNA to provide an estimate of replicating virus. Specifically, vRNA was quantified using the QuantStudio 6 Real-Time PCR System (Applied Biosystems, Waltham, MA). Five microliters vRNA was added in duplicate to a 0.1 mL 96-well MicroAmp fast optical reaction plate (Applied Biosystems, REF# 4346906). For genomic vRNA quantification, the 2019-nCoV RUO Kit (Integrated DNA Technologies, Coralville, IA) was used, according to the manufacturer’s protocol, to target the N1 amplicon of the N gene along with TaqPath 1-Step RT-qPCR Master Mix (Applied Biosystems Waltham, MA). For the subgenomic assay, a forward primer targeting the subgenomic leader sequence and a reverse primer/probe (Integrated DNA Technologies, Waltham, MA) designed to target the N gene, was used along with the TaqPath Master Mix mentioned above. Fifteen microliters of the respective master mix were added to each well and run using the following conditions: 25°C for 2 minutes, 50°C for 15 minutes, 95°C for 2 minutes followed by 40 cycles of 95°C for 3 seconds and 60°C for 30 seconds. In vitro transcribed RNA was quantified and diluted to known copy numbers and used to generate the genomic and subgenomic standard curves. Both genomic and subgenomic viral copy numbers were calculated by plotting Cq values from unknown samples against the respective standard curve. Positive, negative, and non-template controls were analyzed along with each set of samples.

### Isolation of PBMCs

Peripheral blood mononuclear cells (PBMCs) were isolated from whole blood using SepMate-50 Isolation tubes (Stem Cell Technologies, Vancouver, Canada) per the manufacturer’s protocol. Cells were counted using a Cellometer Auto 2000 (Nexcelom, Lawrence, MA), resuspended in Bambanker cell freezing medium (GC Lymphotec, Tokyo, Japan) at approximately 1x10^7^ cells/mL and cryopreserved at -80°C.

### ELISA assays

D-dimer levels in sodium citrate plasma samples were measured via an enzyme-linked immunosorbent assay (ELISA) (Ray Biotech, Peachtree Corners, GA) per the manufacturer’s protocol. Samples were diluted 600,000-fold and plated in duplicate. IL-4 levels in plasma samples were measured using a Monkey IL-4 ELISA kit (abcam, Boston, MA) per the manufacturer’s protocol. Plasma samples were diluted 1:2 and assayed in duplicate along with two replicates of undiluted sample. In modification to the manufacturer’s protocol, the standard/sample incubation time was increased to 2.5 hours. Plates were analyzed using the GloMax Explorer plate reader (Promega, Madison, WI) and GraphPad Prism (GraphPad Software version 9, LaJolla, California). Heatmap was generated using Microsoft Excel. Data was normalized by dividing raw data values from Day 4, (D-dimer only) 6, 14 and 21 by the baseline value for each animal.

Kynurenine and tryptophan levels in plasma were measured using commercially available enzyme-linked immunosorbent assays (Rocky Mountain Diagnostics, Colorado Springs, CO) per the manufacturer’s protocol. The GloMax Explorer plate reader (Promega, Madison, WI) along with GraphPad Prism v9 were used to analyze the plates.

### Quantification of inflammatory cytokines and coagulation biomarkers

BioLegend’s bead-based immunosorbent assays were used to measure inflammatory cytokines in serum (LegendPlex NHP Inflammation Panel, BioLegend, San Diego, CA) and coagulation biomarkers in sodium citrate plasma (LegendPlex Human Fibrinolysis Panel). Serum and plasma samples were diluted 4-fold and 10,000-fold, respectively, and assayed in duplicate. Results were read using a MacsQuant 16 Flow Cytometer (Miltenyi Biotec) and LegendPlex’s online data analysis tool (Qognit). Heatmap was generated using Microsoft Excel. Data was normalized by dividing raw data values from Day 6, 14 and 21 by the baseline value for each animal.

### Flow cytometry analysis

Phenotypic and intracellular cytokine analyses of mononuclear cells (MNC) isolated from blood and bronchoalveolar lavage (BAL) were performed using antibodies against markers listed in S2, S3, and S4 Tables. Briefly, cells were washed and counted with the Cellometer Auto 2000 (Nexcelom Bioscience, Lawrence, MA). Cells were then pelleted and resuspended in Live/Dead stain cocktail (50 μL PBS + 0.5 μL live/dead stain per test) (Fixable Aqua Dead Cell Stain Kit, Invitrogen, Lithuania) and incubated in the dark for 20 minutes. Cells were washed in PBS supplemented with 2% FBS, pelleted, resuspended, and incubated in surface-stain cocktail consisting of 50 μL BD Horizon Brilliant Violet Stain Buffer (BD Bioscience, Franklin Lakes, NJ) plus antibodies (see S2, S3, and S4 Tables) for 20 minutes in the dark. Cells were washed in PBS with 2% FBS, pelleted, then resuspended in 200 μL BD Cytofix/Cytoperm solution (BD Biosciences, Franklin Lakes, NJ) and incubated in the dark for 20 minutes. Cells were washed in 1x BD Perm/Wash Buffer (BD Biosciences, Franklin Lakes, NJ), pelleted, and resuspended in intracellular-staining cocktail consisting of 100 μL 1x BD Perm/Wash Buffer plus antibodies according to S2, S3, and S4 Tables and incubated for 20 minutes in the dark. Finally, cells were washed, pelleted, and resuspended in 200 μL 1x BD Stabilizing Fixative (BD Biosciences, Franklin Lakes, NJ).

### Monocyte cytokine expression

To measure monocyte cytokine expression, MNCs from blood and BAL were washed and counted (Cellometer Auto 2000, Nexcelom Bioscience, Lawrence, MA), pelleted, and then resuspended in DMEM (Gibco, Grand Island, NY) with 5% Anti-Anti (Gibco, Grand Island, NY) at 1x10^6^ cells/mL. Cells were stimulated with lipopolysaccharide at 10 ng/mL (Sigma, St Louis, MO) and incubated with 1 μL/mL Brefeldin-A (BioLegend, San Diego, CA) for 4–6 hours at 37°C, 5% CO_2_. Cells were then stained following the procedure described above with antibodies listed in the Monocyte Panel (S2 Table).

### T cell cytokine expression

MNCs from blood and BAL were counted, washed, pelleted, and resuspended in DMEM with 5% Anti-Anti at 1x10^6^ cells/mL. T cell cytokine expression was measured by stimulating MNCs with cell stimulation cocktail (Biolegend, San Diego, CA) for 4–6 hours at 37°C, 5% CO_2_. To measure T cell responses to SARS-CoV-2 antigens, MNCs from blood and BAL were washed, pelleted and resuspended in DMEM with 5% Anti-Anti and 10% FBS at 1x10^6^ cells/mL followed by overnight stimulation at 37°C, 5% CO_2_ with either cell stimulation cocktail or with one of the following viral peptide pools obtained through BEI Resources, NIAID, NIH: Peptide Array, SARS Coronavirus Nucleocapsid Protein (NR-52419), Spike Glycoprotein (NR-52402), Membrane Protein (NR-53822), or Envelope Protein (NR-53822) along with Brefeldin-A (S5 Table). Cells were stained as described above using the antibodies listed in the T cell panel (S4 Table).

All samples were acquired on a LSRFortessa Cell Analyzer (BD Biosciences, Franklin Lakes, NJ) using BD FACSDIVA 8.0.1 software. Approximately 1x10^6^ cells were acquired from each sample. Data was analyzed using FlowJo version 10.7.1 for MAC (Becton Dickinson and Company, Ashland, OR). SARS-CoV-2 antigen specific T cell responses (Figs 10A, 10B, and S5) were generated using the Matlab based tool cyt3[27]. Data was transformed using arcsin 150. Cytokine expression was measured in FlowJo and, when applicable, applied to cyt3 generated figures. t-distributed stochastic neighbor embedding (tSNE) analysis was performed in FlowJo 10.7.1, nightingale rose plots were generated in R using the ggplot2[28] package, radial plots were generated in Microsoft Excel.

### Histopathology and immunohistochemistry

Zinc-formalin fixed tissues were processed routinely, embedded in paraffin and cut into 5 μm sections for hematoxylin and eosin (H&E), Masson’s Trichrome, or immunohistochemical (IHC) staining.

For H&E staining, tissue samples were collected in Zinc formalin (Anatech, Sparks, NV) and immersion fixed for a minimum of 72 hours before being washed and dehydrated using a Thermo Excelsior AS processor. Upon removal from the processor, tissues were transferred to a Thermo Shandon Histocentre 3 embedding station where they were submersed in warm paraffin and allowed to cool into blocks. From these blocks, 5 μm sections were cut and mounted on charged glass slides, baked overnight at 60°C and passed through Xylene, graded ethanol, and double distilled water to remove paraffin and rehydrate tissue sections. A Leica Autostainer XL was used to complete the deparaffinization, rehydration and routine hematoxylin and eosin stain preparing the slides for examination by a board-certified veterinary pathologist using HALO software (Indica Labs, Albuquerque, NM).

Trichrome staining was performed as previously described with the exception of an additional 10-minute incubation using Weigert’s Iron Hematoxylin Working Solution[29]. Slides were analyzed by a board-certified veterinary pathologist using HALO software for quantification.

For IHC staining, tissue sections were mounted on Superfrost Plus Microscope slides (Fisher Scientific, Carlsbad, CA), incubated for 1 hour at 60°C, and passed through Xylene, graded ethanol, and double distilled water to remove paraffin and rehydrate tissue sections. A microwave was used for heat induced epitope retrieval (HIER). Slides were boiled for 20 minutes in a Tris based solution, pH 9 (Vector Laboratories, Burlingame, CA), supplemented with 0.01% Tween-20. Slides were briefly rinsed in hot, distilled water and transferred to a hot citrate-based solution, pH 6.0 (Vector Laboratories, Burlingame, CA) where they were allowed to cool to room temperature. All slide manipulation from this point forward was done at room temperature with incubations taking place in a black humidifying chamber. Once cool, slides were rinsed in tris buffered saline (TBS) and incubated with Background Punisher (Biocare Medical, Pacheco, CA) for 10 minutes. Slides were then submerged in a solution of TBS supplemented with 0.01% TritonX100 (TBS-TX100) and placed on a rocker platform for two 5-minute washes followed by a TBS rinse before being returned to humidifying chamber to be incubated with serum free protein block (Dako, Santa Clara, CA) for 20 minutes. Mouse anti-Granzyme primary antibody (S6 Table) was then added to the slides and allowed to bind for 60 minutes. Slides were then washed twice with TBS-TX100 and once with TBS. The labeling of the antibody for visualization was done using the MACH3 AP kit (Biocare Medical, Pacheco, CA). Both the MACH3 probe and polymer were incubated for 20 minutes with TBS-TX100 and TBS washes in between. Slides were incubated with permanent red substrate (Dako, Santa Clara, CA) for 20 minutes and placed in TBS to halt the enzymatic reaction.

All other staining was done consecutively with the following method. Slides were incubated with a blocking buffer comprised of 10% normal goat serum (NGS) and 0.02% fish skin gelatin in phosphate buffered saline (PBS) for 40 minutes. This blocking buffer was also used to dilute both primary and secondary antibodies (S6 Table). Primary antibodies were added to slides for 60 minutes. After washing two times with PBS supplemented with 0.02% fish skin gelatin and 0.01% TritonX100 (PBS-FSG-TX100) and once with PBS-FSG, slides were incubated for 40 minutes with a secondary antibody made in goat, raised against the primary host species, and tagged with an Alexa Fluor fluorochrome (488 or 568). The 3 washes (described above) were repeated before DAPI nuclear stain was added for 10 minutes. Slides were mounted using anti-quenching mounting media containing Mowiol (Sigma, St Louis, MO) and DABCO (Sigma, St Louis, MO) and allowed to dry overnight before imaging with a Axio Slide Scanner (Zeiss, Hamburg, Germany). HALO software (Indica Labs Albuquerque, NM) was used for quantification and analysis.

### Detection of neutralizing antibodies in serum

Pseudovirus neutralization testing of serum samples was performed using a SARS-CoV-2.D614G spike-pseudotyped virus in 293/ACE2 cells, with neutralization assessed via reduction in luciferase activity as described[30, 31].

### Statistical analysis

GraphPad Prism (version 9 GraphPad Software, LaJolla California) was used for graphing and statistical analyses. The Kruskal-Wallis test and Dunn’s test for multiple comparisons were used to compare changes in cell frequencies as well as surface marker, cytokine and Granzyme B expression. The Mann-Whitney U test for comparison of means was employed to compare viral loads between PTM and RhM at 1-dpi. A multiple linear regression was conducted in R to compare viral titers, both overall and over time, between PTM and RhM, and corresponding plots were created with ggplot2[28].

## Results

### Viral dynamics

Four male pigtail macaques (PTM) inoculated with SARS-CoV-2 were followed via blood, mucosal swab and bronchoalveolar lavage (BAL) sampling. Two animals were euthanized at 6 days post infection (dpi) and two at 21 dpi (Fig 1A). Quantitative RT- PCR was used to track viral genomic and subgenomic RNA through the course of the study at multiple sites. We detected both genomic and subgenomic SARS-CoV-2 RNA in all four animals throughout the first several days of infection (Fig 1B–1K). One animal, MA27, euthanized at 6-dpi, showed a spike in genomic and subgenomic viral RNA (sgm vRNA) at necropsy in the pharynx (Fig 1F and 1G), with viral levels also beginning to rise in the nasal cavity (Fig 1B and 1C). MA28, euthanized at 21-dpi, showed detectable levels of vRNA in the nasal and rectal mucosa over the course of the study (Fig 1B and 1D). Next we performed a direct comparison of viral titers between our PTM and a cohort of RhM from a recent study of ours[26]. At 1-dpi, PTM have significantly higher titers of virus in the nasal cavity, significantly lower titers in pharynx and no significant difference in titers in the rectal mucosa (Fig 1L and 1M). Comparison of viral titers over time revealed a significant difference in both nasal and pharyngeal sites between the two species (Fig 1N–1S). Interestingly, we also show a significant difference in the rate at which the genomic viral load decreases in the nasal mucosa. Both species show a decline in viral titers over time however, PTM experienced a significantly faster rate of decrease (Fig 1N). The rate of change overtime in pharyngeal sgm vRNA was also found to be significantly different between the two species with the rise in PTM sgm vRNA driven by MA27 (Fig 1R).

### COVID-19 symptoms, pulmonary disease and pathology

The animals in our study were monitored daily for COVID-19 symptoms (S1 Table). Similar to what others have noted in RhM[26], we observed mild COVID-19 symptoms including decreased appetite, soft stool, mild cough and slight increased effort breathing. We found no significant changes in body weight, temperature, or saturation levels of blood oxygen (S1 Fig). Thoracic radiographs were obtained from all animals before infection and weekly thereafter, revealing subtle changes consistent with interstitial pneumonia reflective of mild to moderate COVID-19 (S2 Fig). Postmortem examination at 6-dpi revealed mild-to-moderate SARS-CoV-2-associated pneumonia in one of the two animals, MA27. The pneumonia was characterized by multifocal tan-plum areas of consolidation in the caudal left lung lobe (S3A Fig and S1 Table). At 21-dpi, gross lesions were minimal and only observed in one of two animals, MA28. The lesions noted in this animal were two small, flat tan foci on the dorsolateral aspect of the left caudal lung lobe (S3D Fig and S1 Table). Previous studies in RhM and Cynomolgus macaques (CyM) have demonstrated similar lung pathology with the RM model potentially exhibiting more severe lung lesions than CyM and PTM[16, 32]. The PTM in our study revealed a wider range of lung pathology than that seen in other macaque models. Additional studies with an expanded cohort of PTMs may uncover key pathways of lung pathogenesis that occur with varying levels of disease which is representative of the variation seen in humans.

**Fig 2:**
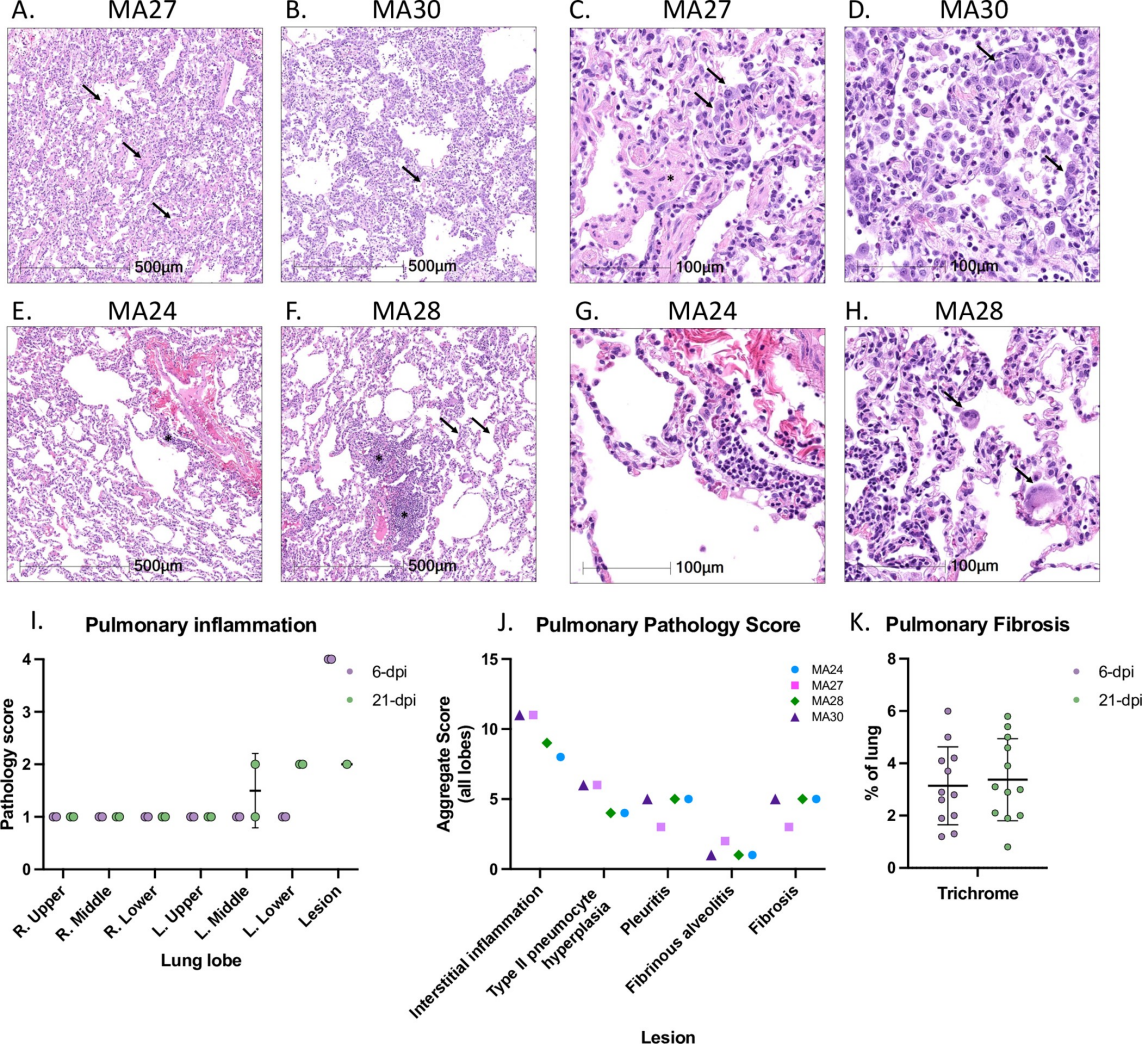
Histopathologic findings in SARS-CoV-2 infected pigtail macaques (PTM). Histopathologic findings at 6- **(**A-D) and 21-dpi (E-H). A and B. At 6-dpi alveolar septa are expanded by inflammatory infiltrate and alveoli contain rafts of fibrin (arrows). C and D. The inflammatory infiltrate is composed of a mixture of histiocytes, lymphocytes, and neutrophils, and alveolar septa are frequently lined by type II pneumocytes (arrows). In severely affected areas, alveoli contain fibrin rafts (C, asterisks). E and F. At 21-dpi, there is residual inflammation composed of perivascular lymphoid aggregates (asterisks), and mild thickening of alveolar septa (arrows). G and H. The residual inflammation is composed predominately of lymphocytes, and in MA28, rare multinucleated giant cells (H, arrows). A quantitative scoring system was used to assess pulmonary pathology in multiple lung sections (I and J). Each lung section was manually quantified for the percent of lung affected by interstitial inflammation (I). Each section was also quantified for the percent of lung affected by several typical SARS-CoV-2 induced pathologic lesions (J). Masson’s Trichrome staining was also performed on the lung sections to quantify fibrosis (K).

Histopathological findings consistent with SARS-CoV-2 associated pneumonia were observed in both animals at 6-dpi. Both animals had an interstitial pneumonia that was localized to regions of the left caudal lung. Regions of interstitial pneumonia were characterized by alveolar septa that were mild to markedly expanded by a mixture of macrophages, lymphocytes, and neutrophils. Alveolar septa were frequently lined by type II pneumocytes (Fig 2C and 2D), and alveoli contained large numbers of alveolar macrophages with rafts of fibrin in more severely affected areas (Fig 2A–2D).

At 21-dpi, minimal-to-mild residual interstitial pulmonary inflammation was observed in both animals. The residual inflammation was composed of perivascular lymphoid aggregates along with mild thickening of alveolar septa (Fig 2E and 2F). The inflammatory infiltrate at this time point was composed predominately of lymphocytes; however, in one animal, MA28, low numbers of multinucleated giant cells were present in alveoli. (Fig 2H).

A comprehensive histopathological scoring system was designed to quantitatively assess pulmonary inflammation and pathogenic changes in all four animals at necropsy (Fig 2I and 2J). Up to seven lung sections were examined from each animal, one from each major lobe (left and right, upper, middle, and lower lobes) as well as a section of a grossly identified lung lesion (no gross lesion was observed in MA24). Each section of lung was manually quantified for the percentage of lung affected by interstitial inflammation (Fig 2I). Lesions were assigned a score for the interstitial inflammation based on the following scale: 0 = 0%, 1 = 0–5%, 2 = 5–10%, 3 = 10–30%, 4 = >30% (inclusive of the upper limit). We also quantified each section of lung for the percentage of lung affected by several SARS-CoV-2 induced pathologic lesions including interstitial inflammation, type II pneumocyte hyperplasia, pleuritis, fibrinous alveolitis, and fibrosis (Fig 2J). Lesions were assigned a score based on the percentage of lung affected using the same scale as described above. For the pulmonary pathology score shown in Fig 2J, the scores of all lobes (seven for 6-dpi and six for 21-dpi) were summated to create an aggregate score for each pathologic lesion in each animal. Significant inflammation and pathology were localized to gross lesions and specific regions of the lung (left middle and left lower).

To assess potential SARS-CoV-2 induced lung fibrosis we performed Masson’s Trichrome staining of sections from each major lung lobe (left and right, upper, middle, and lower lobes, Fig 2K) at necropsy. No appreciable fibrosis was indicated at either 6- or 21-dpi. Overall, the SARS-CoV-2 induced pathology observed in our PTM model is consistent with the mild pathology seen in our recent studies of RhM[15, 26].

### Blood cytokine measures of inflammation

**Fig 3:**
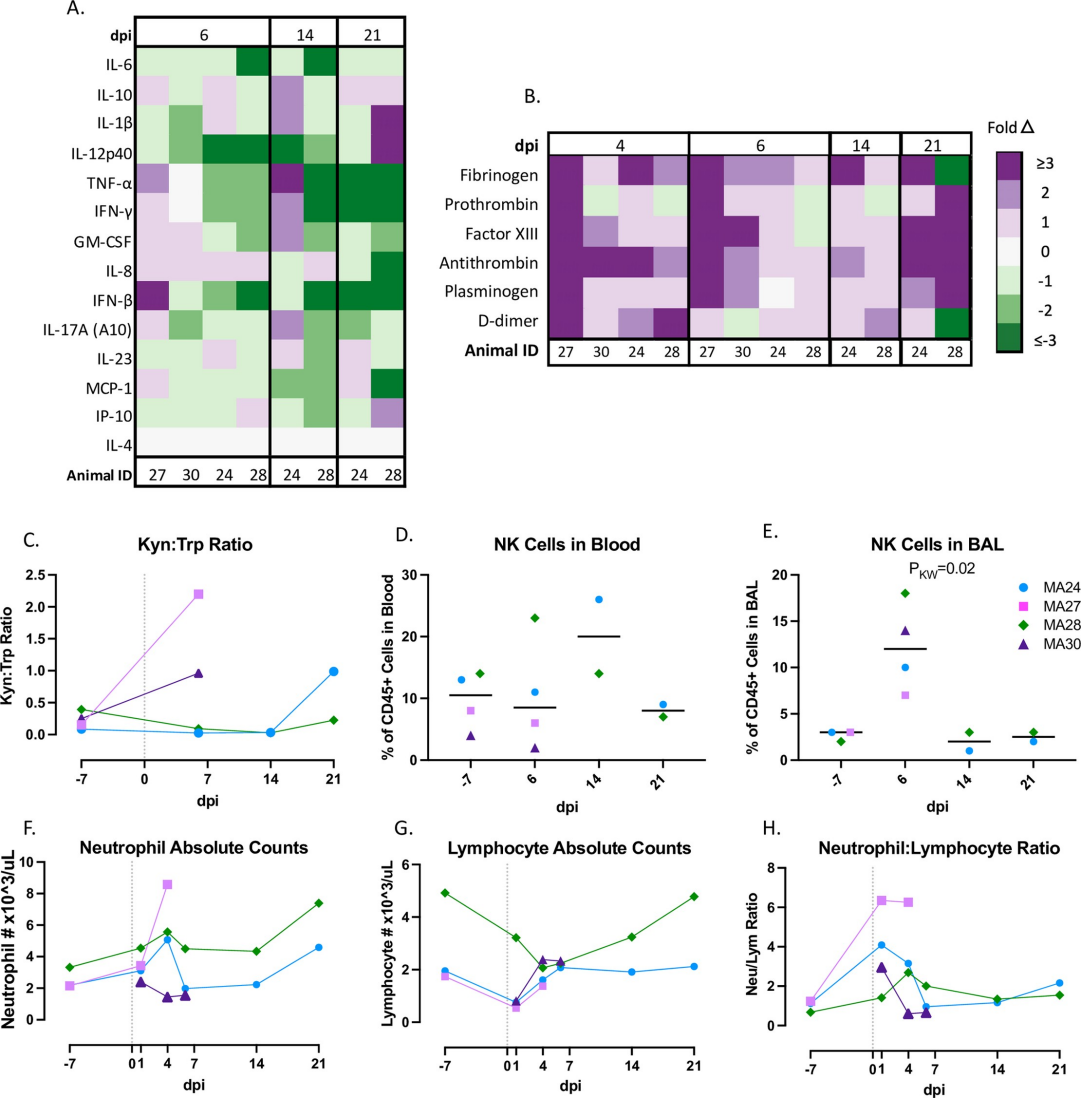
Inflammatory innate immune response in pigtail macaques challenged with SARS-CoV-2. A. Changes in serum/plasma cytokine levels at 6-, 14- and 21-days post SARS-CoV-2 infection (dpi). Data represent fold changes from baseline. B. Changes in coagulation biomarkers in plasma at 4-, 6-, 14- and 21-dpi. Data are fold changes from baseline. C. Ratio of Kynurenine (Kyn) to Tryptophan (Trp) as a measure of indoleamine 2,3-dioxygenase (IDO) activity before and after SARS-CoV-2 infection. D-E. Frequency of Natural Killer (NK, CD45+ CD3- HLA-DR-/lo CD8+) cells in the blood (D) or BAL (E) at baseline and 6-, 14- and 21-days post infection (dpi). Bars represent median. F. Absolute number of neutrophils pre- and post- SARS-CoV-2 infection. G. Absolute number of lymphocytes pre- and post-SARS-CoV-2 infection. H. Changes in neutrophil to lymphocyte ratio before and after SARS-CoV-2 infection. Panels C and D. Baseline: n = 4, Day 6: n = 4, Day 14: n = 2, Day 21: n = 2; Panel E. Baseline: n = 3, Day 6: n = 4, Day 14: n = 2, Day 21: n = 2; Panels F-H. Baseline: n = 3, Day 1: n = 4, Day 4: n = 4 Day 6: n = 4, Day 14: n = 2, Day 21: n = 2. Panels C-H. Day 0 = day of infection. Kruskal-Wallis comparison of overall means (P_KW_) was used to determine significance. P values ≤0.05 reported.

We next measured a panel of cytokines in blood serum after infection. Fluctuations in several inflammatory cytokines, as compared to baseline, were found throughout the study. Interleukin-8 (IL-8), a neutrophil chemoattractant, was the most consistently increased cytokine at 6-dpi whereas IL-6 and IL-12-p40 decreased in all animals at day 6 (Fig 3A). Interestingly, MA27 had a stronger inflammatory cytokine response at 6-dpi compared to the other three animals, as exemplified by increases in several cytokines, including IL-10, IFN-**γ**, GM-CSF, IL-8, IL-17A, MCP-1 and most notably, TNF-⍺ and IFN-β. As stated previously, this animal had increasing viral loads at 6-dpi suggesting a possible link between the intensity of the inflammatory response and the level of replicating virus. Animal MA28, which exhibited consistently high genomic vRNA levels in both nasal and rectal swabs through 21-dpi, showed a rise IL-10, IL-1β, IL-12p40 and IP-10 serum levels at necropsy (21-dpi). We also measured levels of IL-4, an anti-inflammatory cytokine, in plasma before and after infection. The IL-4 concentration in our samples was below the level of detection of the assay and is therefore represented in the figure as no change occurring throughout the course of the study.

### Markers of coagulopathy

Complications related to coagulopathy have been reported in humans with severe COVID-19 disease, with highly elevated levels of D-dimers shown to be a particular correlate of disease severity[33, 34]. To examine whether PTM recapitulate this phenotype, we measured multiple biomarkers of coagulation in blood (Fig 3B), including fibrinogen, prothrombin, factor XIII, antithrombin, plasminogen, and D-dimers. We found nearly universal increases in coagulation biomarkers in the first week of infection. Specifically, we noted increased D-dimer levels in all four animals at 4-dpi, with MA27 and MA28 exhibiting a greater than 3-fold increase relative to baseline before resolving to near baseline levels. Interestingly, several biomarkers (prothrombin, factor XIII, antithrombin, and plasminogen) began to rise again at 21-dpi.

### Kynurenine tryptophan pathway

Pro-inflammatory cytokines, specifically interferon gamma-**γ** (IFN-**γ**), promote the kynurenine (Kyn) pathway (KP) of tryptophan (Trp) catabolism[35]. Recent studies in humans hospitalized with COVID-19 suggest that the Kyn:Trp ratio positively correlates with disease severity[36]. We measured the Kyn:Trp ratio in plasma at baseline, and days 6, 14 and 21 (Figs 3C, S4A and S4B). Again, MA27 showed the greatest increase in the Kyn:Trp ratio at 6-dpi possibly providing another biomarker of the more severe disease course seen in this animal.

### NK cells

The initial immune response to SARS-CoV-2 infection involves the intricate interplay between the cells of the innate immune system. Natural killer (NK) cells are cytotoxic lymphocytes that often play a key role in the early defense against viral infections. Studies of hospitalized COVID-19 patients show that decreases in circulating NK cells correlate with disease severity[37, 38]. Here, we measured the percentage of NK cells (defined as CD45+ CD3- HLA-DR-/lo CD8+) in both the blood and bronchoalveolar lavage fluid (BAL) at baseline, and days 6-, 14-, and 21-post infection (Fig 3D and 3E). We did not find significant changes in peripheral NK cells in our study. However, MA28 and MA24 had slight increases in circulating NK cells at day 6 and day 14, respectively. Flow cytometry analysis of BAL indicated an increase in infiltrating NK cells in the lung at 6-dpi in all four animals.

### Neutrophil to lymphocyte ratio

A high incidence of neutrophilia coupled with lymphocytopenia has been reported in COVID-19 patients[38, 39]. Animals MA24, MA27, and MA28 all experienced neutrophilia and lymphocytopenia during the course of the study. However, these changes were mild, and values largely remained within normal limits. Pre-infection data on these cells were not available for MA30 (S1 Table). The neutrophil to lymphocyte ratio (NLR) has been identified as an important predictor of disease severity in human patients[40]. Thus we measured the NLR at baseline and 6-, 14- and 21-dpi. Interestingly, the highest NLR at 1- and 4-days post infection was noted in MA27 (Fig 3F–3H). These data are consistent with the increasing viral titers noted at 6-dpi, the increased levels of inflammatory cytokines and D-dimers, as well as the elevated K:T ratio observed in MA27. Each potentially correlate with or contribute to the more severe lung pathology noted in this animal at necropsy.

### SARS-CoV-2 infection and macrophage pulmonary infiltration

**Fig 4:**
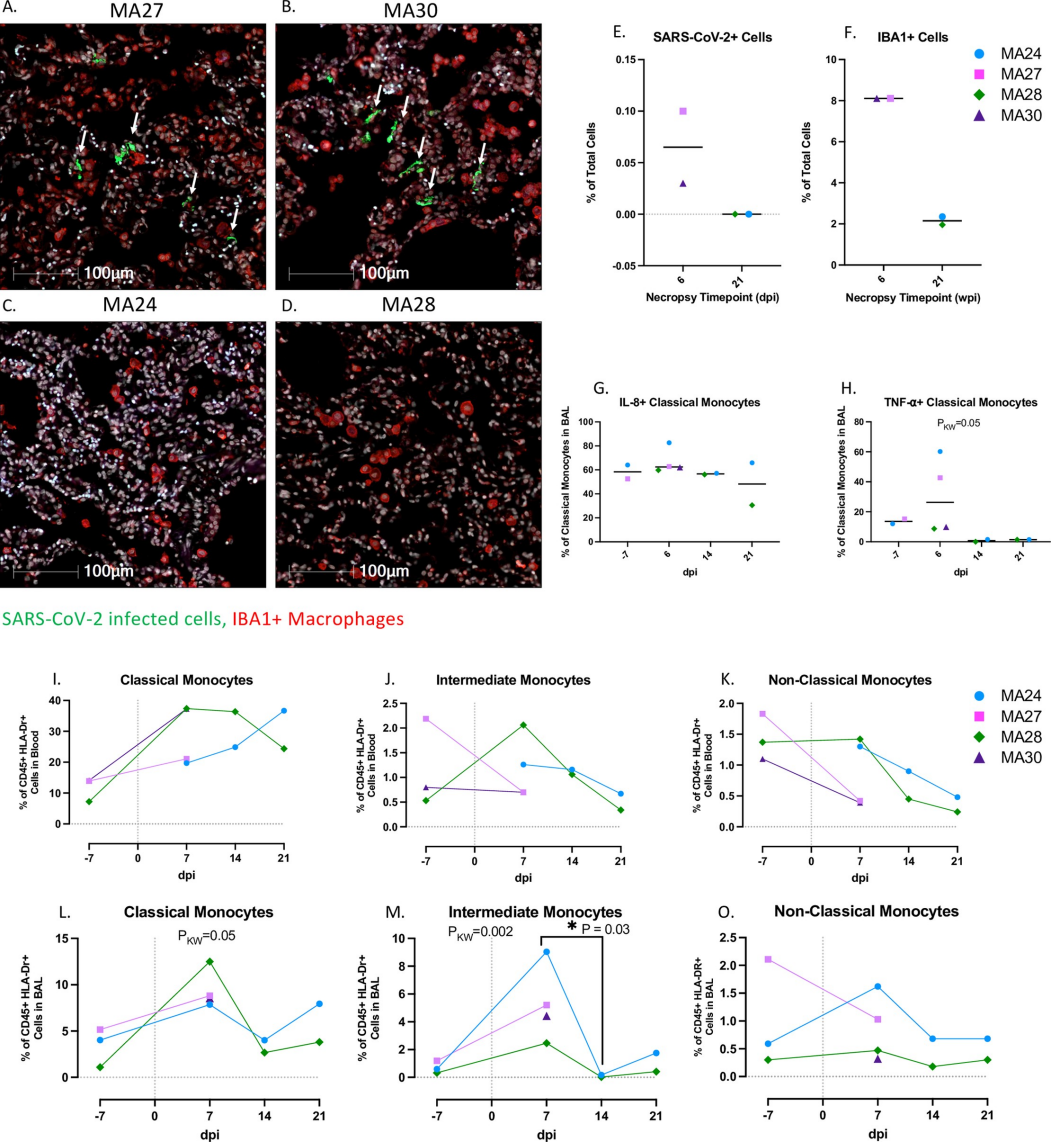
Pulmonary SARS-CoV-2 infection and macrophage/monocytes in the lung and blood. A-D. SARS-CoV-2 infection and macrophage infiltration in the lungs of pigtail macaques at 6- (A and B) and 21- days post infection (dpi, C and D). DAPI = White, Green = SARS-CoV-2, Red = IBA1, Blue = Autofluorescence. E and F. Percentage of SARS-CoV-2 infected cells (E) and IBA1+ macrophages (F) in the lung at necropsy. Bars represent median. G and H. Frequency of IL-8 (G) and TNF-⍺ (H) expressing classical monocytes (CD45+ HLA-DR+ CD14- CD16+) in BAL. I-O. Frequencies of Classical (I and L) intermediate (CD45+ HLA-DR+ CD14+ CD16+) (J and M), and non-classical monocytes (CD45+ HLA-DR+ CD14- CD16+) (K and O) in the blood and BAL before and after SARS-CoV-2 infection. Day 0 = day of infection. Panels I-O. Baseline (day -7): n = 3, Day 6: n = 4, Day 14: n = 2, Day 21: n = 2. Kruskal-Wallis comparison of overall means (P_KW_) and Dunn’s Multiple comparisons (designated by line, P_D_) tests used to determine significance. P values ≤0.05 reported.

Fluorescent immunohistochemistry of the lung for SARS-CoV-2 nucleoprotein identified small clusters of SARS-CoV-2 infected cells, predominately lining the alveolar septa, in both animals sacrificed at 6-dpi (Fig 4A, 4B and 4E). COVID-19 disease is commonly characterized by pulmonary infiltration of inflammatory immune cells[41]. Innate cells, particularly monocytes/macrophages are considered important mediators of disease progression[42]. At 6-dpi, the alveoli contained large numbers of IBA1+ macrophages (Fig 4A, 4B and 4F). By 21-dpi, macrophage numbers were greatly reduced and no SARS-CoV-2+ cells were detected in either MA24 or MA28 (Fig 4C–4F).

**Fig 5:**
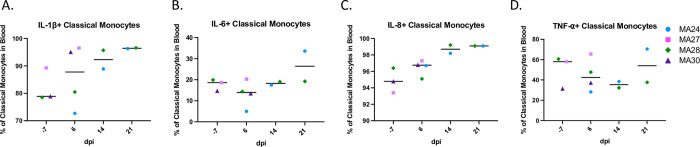
Monocyte cytokine response in the blood of pigtail macaques challenged with SARS-CoV-2. A-D. Frequency of IL-1β (A), IL-6 (B), IL-8 (C) and TNF-⍺ (D) expressing classical monocytes (CD45+ HLA-DR+ CD14- CD16+) in the blood. Bars represent median. Day 0 = day of infection. Baseline (-7): n = 3, Day 6: n = 4, Day 14: n = 2, Day 21: n = 2.

Flow cytometry showed increases in CD14+ CD16- classical monocytes in both the blood and BAL at 6-dpi (Fig 4I and 4L) and an increase in CD14+ CD16+ intermediate monocytes (Fig 4M) in BAL at 6-dpi. Heterogeneous fluctuations of circulating intermediate and CD14- CD16+ non-classical monocytes occurred throughout the study (Fig 4J and 4K). MA27 and MA24 showed increases in inflammatory cytokine, tumor necrosis factor-⍺ (TNF-⍺)-expressing classical monocytes in the BAL at 6-dpi (Fig 4H). Interleukin-1β (IL-1β) and IL-6 are key inflammatory cytokines involved in the pathophysiology of COVID-19 disease in humans[43, 44]. Here, we noted increases in IL-1β expression in peripheral classical monocytes throughout the study (Fig 5A). As previously shown in Fig 3A, serum levels of the inflammatory cytokine IL-6 remained low after SARS-CoV-2 infection. We also found that peripheral monocyte expression of IL-6 remained relatively stable post infection, with only one animal, MA24, showing an increase at days 14 and 21 as compared to day 6 (Fig 5B). Lastly, an upward trend in neutrophil chemoattractant (IL-8+) expressing classical monocytes was observed throughout infection, although this trend was not statistically significant (Fig 5C).

### Peripheral T cell responses

**Fig 6:**
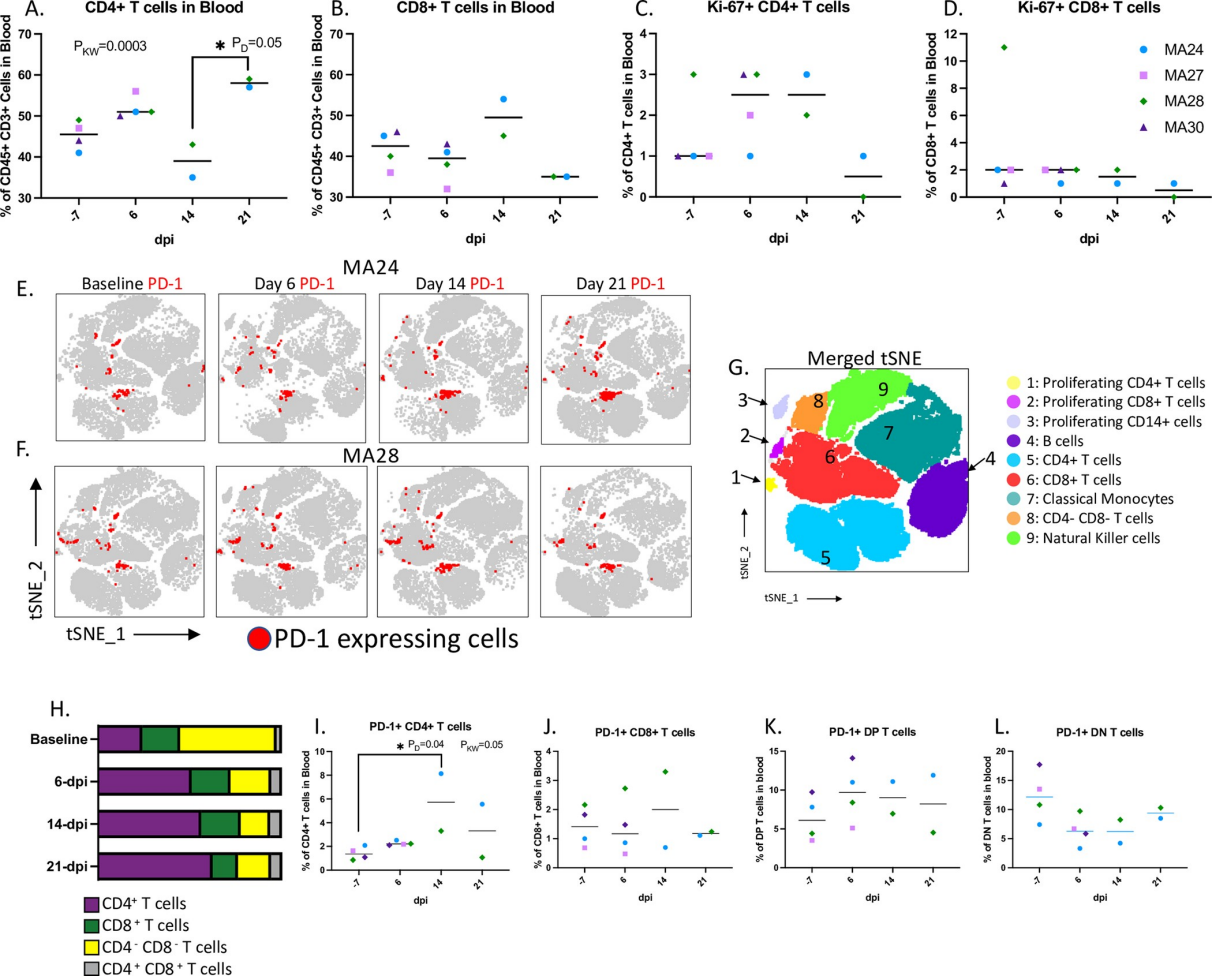
T cells in the blood. A-B. CD4+ (A) and CD8+ (B) T cell frequencies in the blood before and 6-, 14-, and 21-days post SARS-CoV-2 infection. C-D. Changes in Ki-67 expressing CD4+ (C) and CD8+ (D) T cells. Bars represent median E. tSNE plots displaying changes in PD-1 expression (red) in peripheral CD45+ cells overtime. MA24 (E) and MA28 (F) displayed as representative animals. G. Merged tSNE indicating phenotype of the tSNE defined cell populations in E and F. H. Average changes in the percentages of CD4+, CD8+, CD4- CD8- (DN) and CD4+ CD8+ (DP) T cells within the total PD-1+ CD3+ cell population. I-L. Frequency of PD-1+ expressing CD4+ (I), CD8+ (J), DP (K) and DN (L) T cells in the blood. Bars represent median. Kruskal-Wallis comparison of overall means (P_KW_) and Dunn’s Multiple comparisons (designated by line, P_D_) tests used to determine significance. P values ≤0.05 reported. Baseline (-7): n = 4, Day 6: n = 4, Day 14: n = 2, Day 21: n = 2.

Understanding the role of the adaptive immune response to SARS-CoV-2 infection is a key component to the development of effective vaccines and treatment options for COVID-19. Using flow cytometry, we measured changes to T cell populations in both the blood and BAL at baseline and 6-, 14-, and 21-dpi. CD3+ T cell fluctuations in the blood were driven by CD4 T cells which showed levels increasing significantly between days 14- and 21-pi (Fig 6A). As the percentage of CD4 T cells rise and fall over the course of the study, we observed the opposite pattern in the percentage of cytotoxic CD8 T cells (Fig 6B). We found increases in Ki-67+ CD4 T cells at 6-dpi (MA27, MA28 and MA30) and 14-dpi (MA24 and MA28) indicating increased CD4 T cell proliferation (Fig 6C and 6D). Increases in expression of the T cell exhaustion marker, PD-1, have been noted in a number of studies involving human COVID-19 patients[45–47]. Here we found a significant increase in PD-1+ CD4 T cells at 14-dpi (Fig 6I). Interestingly, we saw a decrease in the percentage of CD4 T cells at this same timepoint.

We then used tSNE analysis to show changes in PD-1 expressing cell populations over the course of the study (Fig 6E–6H). At baseline, CD4- CD8- (double negative) T cells made up the greatest proportion of PD-1+ CD3+ T cells (Fig 6H). Beginning at day 6-pi, CD4 T cells made up the majority of PD-1 expressing cells, with only one animal, MA28, showing increases in PD-1 expressing cytotoxic T cells at 6 and 14-dpi (Fig 6J).

### Pulmonary T cell responses

**Fig 7:**
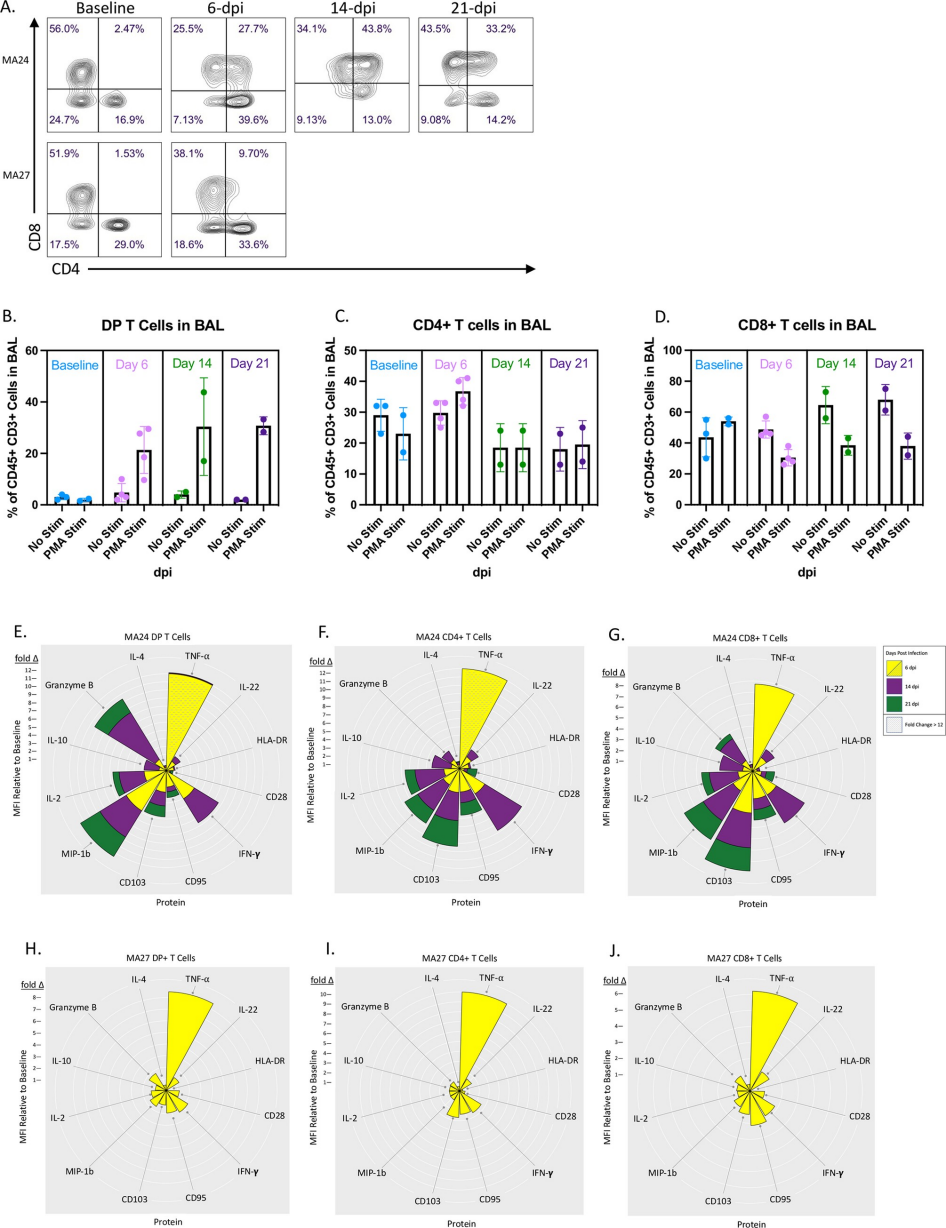
Adaptive T cell responses in the BAL. A. Representative flow cytometry plots showing changes in CD4 and CD8 expression in PMA/ionomycin stimulated CD3+ cells in BAL. Two animals shown (MA24 euthanized at 21-dpi, MA27 euthanized at 6-dpi). B-D. Effect of PMA/ionomycin on the frequency of CD4+ CD8+ (DP, B), CD4+ (C) and CD8+ (D) T cells in BAL before and 6-, 14-, and 21-days post SARS-CoV-2 infection. Bars represent mean and standard deviation. E-J. Nightingale Rose Plots (NRPs) showing fold changes in cytokine and surface protein expression compared to baseline (MFI). Yellow = 6-dpi, Purple = 14-dpi, Green = 21-dpi. Size of petals represents magnitude of increase in expression. Distance from one white ring to the next is a 1-fold change. A decrease in expression is represented by a petal size less than the distance between two rings. Two animals shown (MA24 euthanized at 21-dpi, MA27 euthanized at 6-dpi). At 6-dpi, MA24 DP T cell TNF-⍺ MFI is 24x baseline and CD4+ T cell TNF-⍺ MFI is 50x baseline. Graph cutoff is set to a 12-fold change. Panels B-D. Baseline: n = 3 (No Stimulation (Stim)) and n = 2 (Stim), Day 6: n = 4 (No Stim) and n = 4 (Stim), Day 14: n = 2 (No Stim) and n = 2 (Stim), Day 21: n = 2 (No Stim) and n = 2 (Stim).

We next sought to characterize the dynamics of pulmonary T cell populations over the course of infection by examining the frequency as well as cytokine and surface protein expression before SARS-CoV-2 infection, and at days 6-, 14-, and 21-post viral challenge. Using PMA stimulation, we noted increased frequencies of CD4+/CD8+ double positive (DP) T cells after viral challenge which remained elevated throughout the study (Fig 7A and 7B) ((Median DP T cells as a percentage of CD3+ T cells: Baseline: 2% (n = 2), 6-dpi: 23% (n = 4), 14-dpi: 30% (n = 2), 21-dpi: 31% (n = 2)). We also examined fold changes in surface protein and cytokine expression among the DP, CD4 and CD8 single positive T cell populations as compared to baseline in two of the animals, MA24 and MA27 (Fig 7E–7J). Both MA24 (euthanized at 21-dpi) and MA27 (euthanized at 6-dpi) showed large increases among all three T cell subsets in TNF-⍺ expression at 6-dpi. At fourteen days post infection, MA24 showed increased expression of Granzyme B in both the DP and CD8 T cell populations. Interestingly, it was the DP T cell population which showed the greatest fold increase in Granzyme B over baseline indicating the cytotoxic potential of this DP T cell population.

**Fig 8:**
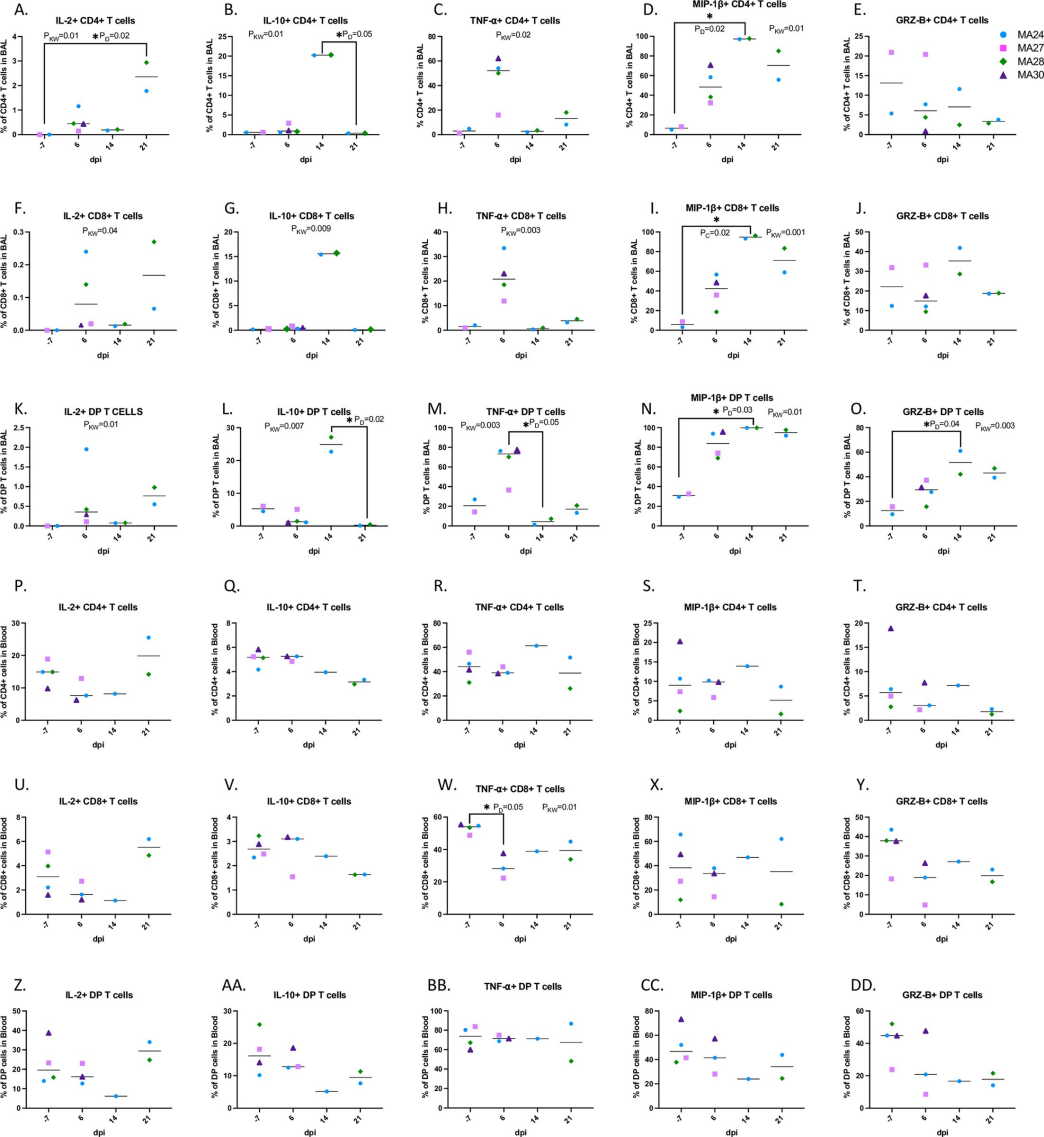
Changes in T cell cytokine expression in the lung (A-O) and blood (P-DD). PMA/ionomycin stimulated CD4+ T cells (BAL: A-E, Blood: P-T), CD8+ T cells (BAL: F-J, Blood: U-Y), and CD4+ CD8+ (DP) T cells (BAL: K-O, Blood: Z-DD). Bars represent median. Kruskal-Wallis comparison of overall means (P_KW_) and Dunn’s Multiple comparisons (designated by line, P_D_) tests used to determine significance. P values ≤0.05 reported. Panels A-O. Baseline (-7): n = 2, Day 6: n = 4, Day 14: n = 2, Day 21: n = 2. Panels P-DD. Baseline (-7): n = 4, Day 6: n = 3, Day 14: n = 1, Day 21: n = 2.

We also compared the activity of each T cell subtype within the same time point of infection (Fig 8A–8O). Prior to infection, DP T cells showed higher TNF-α, IL-10, MIP-1β, and IL-22 expression than traditional CD4 and CD8 T cells, suggesting that these cells may potentially perform a non-specific function in the pulmonary immune response[48]. After viral challenge, we found higher frequencies of Granzyme B expressing DP T cells compared to CD4 T cells and, most notably, CD8 T cells at each timepoint post infection (Fig 8E, 8J and 8O). We found significant increases in CD4 T cells expressing IL-2, IL-10, TNF-⍺ and MIP-1β (Fig 8A–8E). At 14-dpi, we noted a significant increase in MIP-1β expressing CD8 T cells (Fig 8I) along with significant changes in IL-2, IL-10 and TNF-⍺ expression. DP T cells also showed increased activity post viral challenge with significant increases in the frequency of IL-10, TNF-α, MIP-1β and Granzyme B expressing cells. Taken together, these findings show that the DP T cell population has functions which overlap with both CD4 and CD8 T cells[48]. We speculate that these cells are major histocompatibility complex class II (MHC-II) restricted CD4 T cells which upregulate CD8 upon activation, generating the described DP T cell population which has greater cytotoxic potential than traditional CD4 T cells. Pulmonary infiltrating cytotoxic CD4 T cells potentially aid CD8 T cells in viral clearance and are a unique aspect of COVID-19 disease[25].

### Peripheral T cell cytokine responses

We next performed T cell subtype cytokine analysis in the blood using the same panel we used for BAL (Fig 8P–8DD). We found that the dynamic changes in T cell activity occurred mainly in the BAL (Fig 8A–8O) with very few changes occurring in the periphery (Fig 8P–8DD). As COVID-19 is a respiratory disease, these results are not surprising. The only significant change noted in the blood was a decrease in TNF-⍺ expressing CD8+ T cells at 6-dpi (Fig 8W). Interestingly, we see an increase in TNF-⍺ expressing CD8+ T cells in the lung at this same timepoint.

### CD4 T cell and Granzyme B expression in the lungs

**Fig 9:**
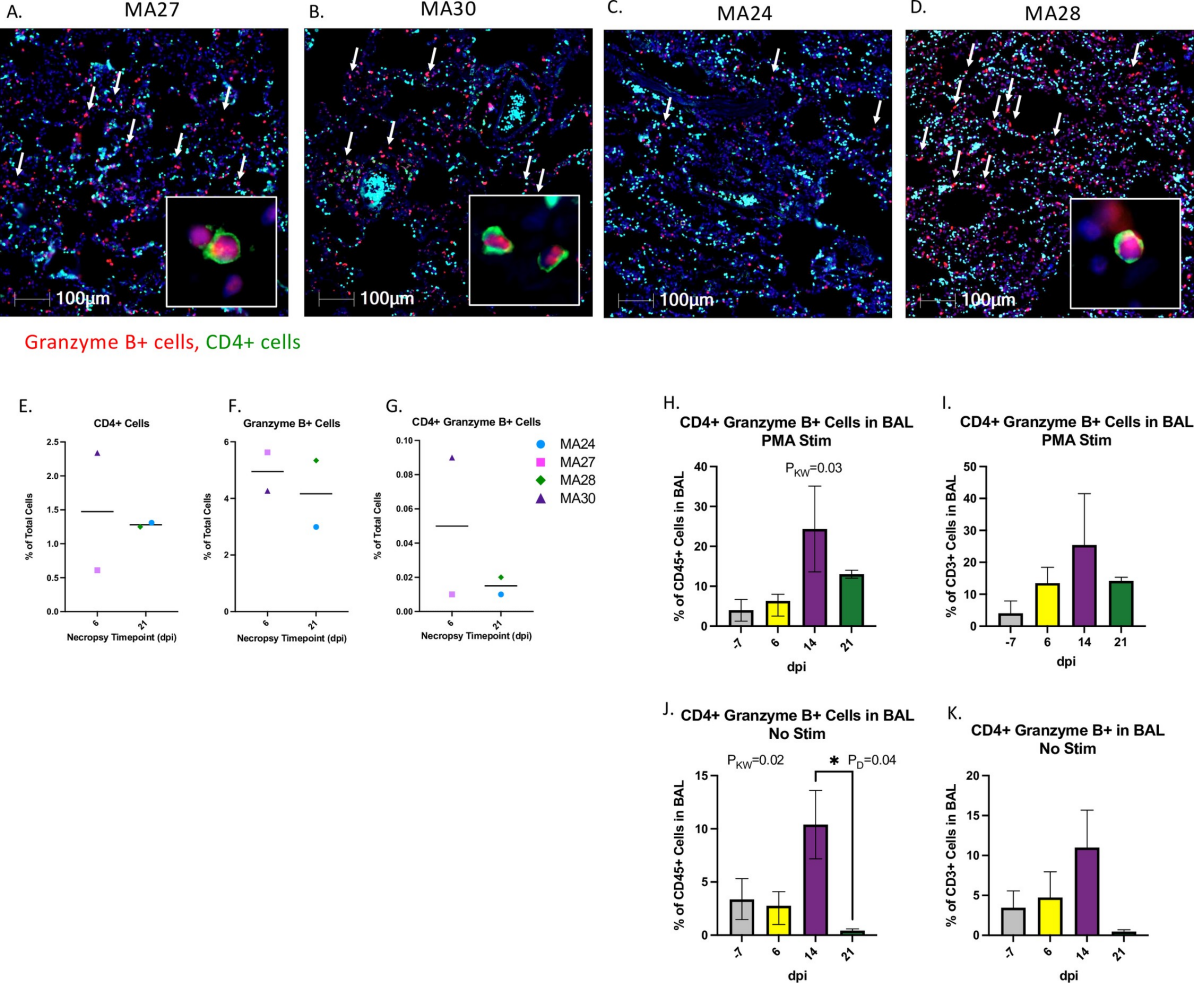
**CD4 and Granzyme B expression in the lungs of SARS-CoV-2 infected macaques at 6- (A and B) and 21-days post infection (dpi, C and D**). At 6-dpi, MA27 (A) and MA30 (B) the lungs are infiltrated by large numbers of Granzyme B positive cells (red, arrows). Insets: Rare CD4+ cells (green) exhibit granzyme expression. At 21-dpi, MA24 (C) exhibits low numbers of Granzyme B positive cells (red, arrows) compared to MA28 (D) and the two, 6-dpi animals (A and B). DAPI = Blue, Green = CD4, Red = Granzyme B. E-G. Percentage of CD4+ (E), Granzyme B+ (F) and CD4+ Granzyme B+ (G) cells in the lung at necropsy. Bars represent median. H-K. CD4+ Granzyme B+ T cells (CD45+ CD3+ CD4+ Granzyme B+) in BAL at Baseline (-7) and 6-, 14- and 21-dpi as a percentage of CD45+ cells (H and J) and CD3+ cells (I and K). H and I. Mononuclear cells, isolated from BAL, were stimulated with PMA/ionomycin for 4–6 hours. Bars represent median and standard deviation. Kruskal-Wallis comparison of overall means (P_KW_) and Dunn’s Multiple comparisons (designated by line, P_D_) tests used to determine significance. P values ≤0.05 reported. Panels H-K. Baseline: n = 3 (No Stimulation (Stim)) and n = 2 (Stim), Day 6: n = 4 (No Stim) and n = 4 (Stim), Day 14: n = 2 (No Stim) and n = 2 (Stim), Day 21: n = 2 (No Stim) and n = 2 (Stim).

Fluorescent Immunohistochemistry (IHC) identified cytotoxic CD4 T cells (CD4+ Granzyme B+) in the lungs of all four PTM at necropsy (Fig 9). We detected large numbers of infiltrating Granzyme B positive cells in the lungs of MA27 and MA30 (euthanized at 6-dpi) along with rare cytotoxic T cells (Fig 9A, 9B, 9F and 9G). At 21-dpi, MA24 (Fig 9C) showed low numbers of Granzyme B+ cells compared to MA28 (Fig 9D) and the other two animals which were euthanized at 6-dpi. Cytotoxic CD4 T cells were detected in the lung of MA28 and, with less frequency, in MA24 (Fig 9G).

We then used flow cytometry to measure cytotoxic CD4 T cells in BAL (CD45+CD3+CD4+Granzyme B+). To mirror the IHC analysis, we did not exclude CD8+ cells from our cytotoxic CD4+ population. Mononuclear cells were incubated with or without PMA stimulation cocktail for 4–6 hours and cytotoxic CD4 T cells were measured as a percentage of CD45+ cells (Fig 9H and 9J) and CD3+ cells (Fig 9I and 9K) in both the stimulated and unstimulated conditions. We noted a considerable increase in cytotoxic CD4 T cells in BAL at 14-dpi.

### SARS-CoV-2 peptide specific T cell response in the lung 21-days post infection

**Fig 10:**
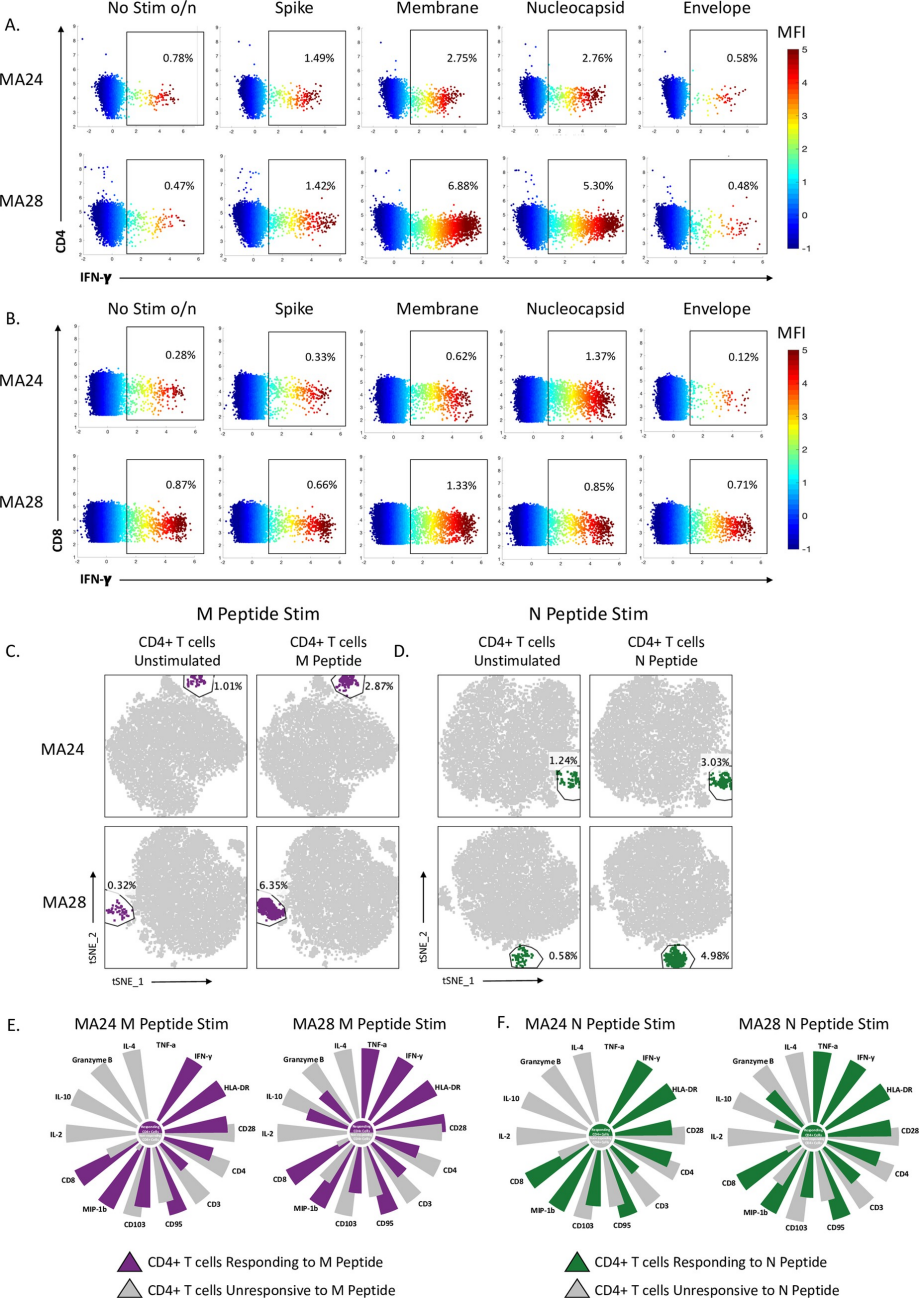
SARS-CoV-2 peptide specific T cell response in the lung 21-days post infection. Two animals shown (MA24 and MA28 euthanized at 21-dpi) A and B. Flow cytometry dot plots showing CD4+ (A) and CD8+ (B) T cell Interferon-**γ** (IFN-*γ*) response to overnight SARS-CoV-2 peptide (spike, membrane, nucleocapsid and envelope) stimulation. No stim o/n = cells incubated overnight without stimulation. Heatmap represents arcsin transformed MFI values. C and D. tSNE plots of CD4+ T cells showing an expansion in cells following overnight peptide stimulation. M = SARS-CoV-2 membrane peptides (C), N = SARS-CoV-2 nucleocapsid peptides (D). E and F. Radial bar plot comparing MFI values of the expanded CD4+ T cell population gated on in Panels C and D to the unchanged CD4+ population within the same tSNE plot. Representative animals MA24 and MA28 (euthanized at 21-dpi). The higher MFI value is set to 100 and the percent difference is calculated between the higher and lower MFI values. Size of the petals represents this analysis.

Mononuclear cells, isolated from BAL, were incubated overnight with SARS-CoV-2 peptides and analyzed by flow cytometry. We detected specific CD4 T cell responses against SARS-CoV-2 that localized to the lung 21 days after viral infection. Specifically, we identified CD4 T cell responses to membrane, nucleocapsid and to a lesser degree, spike peptides (Fig 10A). CD8 T cell responses against the virus were also noted, but at lower frequencies (Fig 10B). In FlowJo, we gated on the CD4 T cell population and applied tSNE analysis to identify and characterize virus specific CD4 T cells responding to membrane and nucleocapsid viral peptides (Fig 10C and 10D). tSNE analysis revealed a unique cluster of CD4 T cells that responded to stimulation. In this population of responding cells (Fig 10E and 10F), we noted increased expression of CD8 and HLA-DR, indicating cell activation. Increased expression of inflammatory cytokines and chemokines was also detected in the antiviral CD4 T cells. We noted a decrease in Granzyme B expression suggesting that the antigen specific CD4 T cells have reduced cytotoxic capacity, unlike the DP T cells (cytotoxic CD4) described previously (Figs 7, 8 and 9). As expected, antiviral CD4s have increased CD95 expression reflecting a memory phenotype. Numerous studies of SARS-CoV-2 convalescent humans have described antiviral T cells with a relative predominance of CD4 T cells[49, 50]. These antiviral responses are most often noted in the blood. In our study, we were unable to detect antigen-specific T cell responses in the blood 21 days after viral infection (S5 Fig). Taken together, our data provide a valuable addition to the data from humans and may suggest important roles for antiviral CD4 T cells in the pulmonary compartment.

### Humoral immune responses

**Fig 11:**
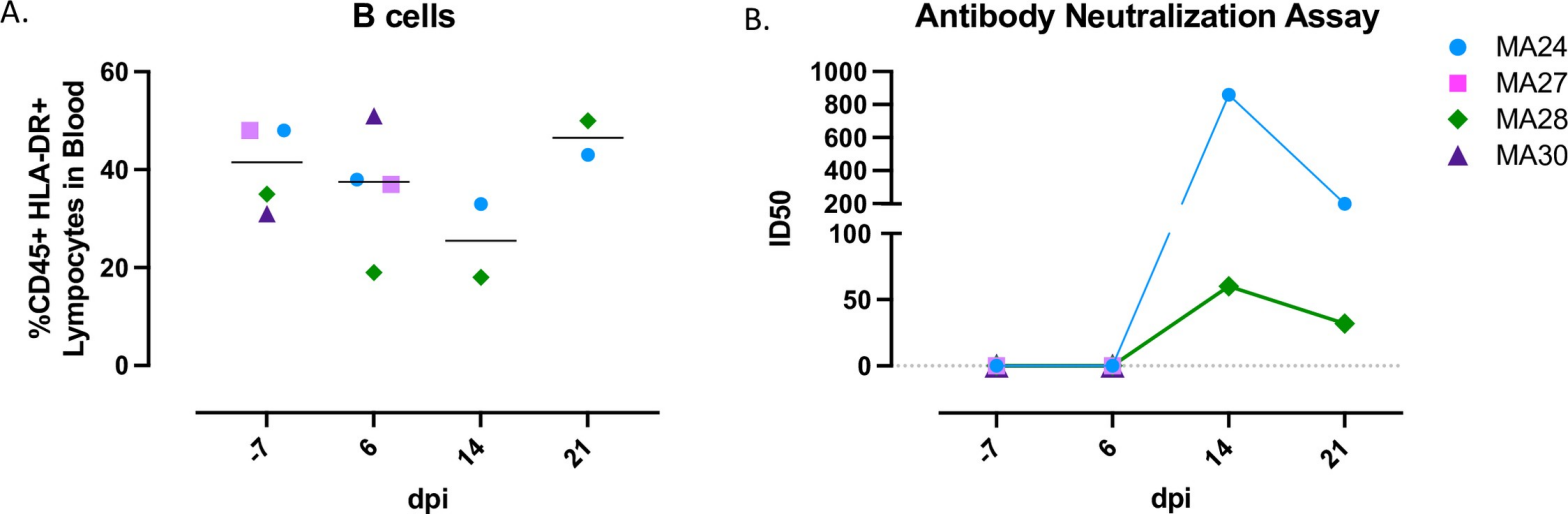
Humoral immune response in SARS-CoV-2 infected pigtail macaques. A. B cell frequencies in the blood before and 6-, 14-, and 21-days post (dpi) SARS-CoV-2 infection. Bars indicate median. (B) Pseudovirus neutralization assay showing serum antibody levels against SARS-CoV-2 using HEK 293T/ACE2 cells. Baseline (-7): n = 4, Day 6: n = 4, Day 14: n = 2, Day 21: n = 2.

Using flow cytometry, we measured B cell kinetics in the blood at baseline and days 6-, 14- and 21-post infection (Fig 11A). We did not detect any significant changes in the percentage of peripheral B cells over the course of the study. We next tested serum from infected animals for neutralizing antibodies using a pseudovirus assay. Unsurprisingly, no neutralization was detected at 6-dpi in any sample, including the animals euthanized at that time point. By 14-dpi, neutralizing antibody responses were detectable in both MA24 and MA28 with responses decreasing by 21-dpi (Fig 11B).

## Discussion

The novel coronavirus SARS-CoV-2 has caused a global pandemic with little precedent. As of the time of submission, this virus has infected nearly 270 million individuals worldwide and killed over five million, including almost 800,000 in the United States. Illness caused by this virus, termed COVID-19, ranges from asymptomatic[51, 52] to flu-like symptoms to severe pneumonia[53, 54]. In the most severe cases, patients have experienced acute respiratory distress syndrome (ARDS) and death[55]. It has also become apparent that a number of surprising symptoms can be associated with SARS-CoV-2 infection, including: coagulopathy, thrombosis, kidney failure and chronic respiratory/neurological issues that seemingly persist well beyond viral clearance[43, 56–63]. Although several highly effective vaccines have been created to combat the COVID-19 pandemic[64–66], billions of individuals remain unvaccinated worldwide. Furthermore, the emergence of new viral variants with enhanced transmissibility[67–70] and the ability to infect even the vaccinated[71] (though this population is overwhelmingly protected from severe disease[72–74]) suggest that this virus will persist indefinitely. Barring the development and mass deployment of vaccines capable of inducing sterilizing immunity, an exceedingly difficult task, intense research focus must remain to decipher disease mechanisms so those that do become infected can be treated.

Critical to both understanding and treating the broad spectrum of disease sequelae caused by SARS-CoV-2 is the development of animal models that faithfully recapitulate COVID-19. Animal models allow timed infection and euthanasia along with extensive sample collection that are not possible during human infections. Rhesus macaques (RhM), cynomolgus macaques (CyM), African green monkeys (AGM), baboons and marmosets have all been used to achieve this goal[14–18, 75]. To date, none of these models consistently recapitulate severe COVID-19 disease but some data suggest AGM may exhibit more severe disease than the others[14, 15]. When infected with simian immunodeficiency virus (SIV), pigtail macaques (PTM) exhibit rapid and severe disease relative to RhM and CyM, including rapid destruction of the CD4 immune compartment, severe gastrointestinal disease, and complications related to coagulopathy[21–23, 76–78]. Many of these disease features are also relevant to severe COVID-19 disease[56–61, 79–81]. PTM have been successfully used to model other viral infections and in some cases show severe disease. Compared to other macaque models, the PTM pregnancy model of Zika infection is more likely to show congenital disease with implications of fetal brain injury similar to that seen in humans[82]. PTM are also susceptible to influenza infection and show a strong persistent immune response to infection[83]. A recent report demonstrated that a related species of pigtail macaques (*Macaca leonine*) showed an abbreviated period of SARS-CoV-2 viral replication but possibly more severe disease than RhM[84]. Thus, PTM may be a reasonable model for severe disease and used to test novel therapeutics and vaccines to prevent disease. Until recently, the northern PTM (*Macaca leonina*), mentioned above, was considered a subspecies of the southern PTM (*Macaca nemestrina*), the species used in our study. They are now considered two distinct species. Unfortunately, PTM in general are not as widely used for biomedical research as are other species such as RhM, so we cannot be certain if our data are representative of northern PTM or any other related species.

We infected a small cohort of PTM with SARS-CoV-2 through a combination of intratracheal and intranasal instillation. Animals were tracked for viral replication in multiple sites, for immune dynamics in blood and bronchoalveolar lavage cells, and for innate and other markers of disease in blood and tissues. We identified a range of disease severity, even in our small cohort, with one animal euthanized at six days post infection showing more severe pulmonary lesions than the rest. Interestingly, multiple early indicators that are consistent with a more severe disease course in humans, were also detected in this animal, including: viral titer, an elevated neutrophil to lymphocyte ratio, elevated kynurenine to tryptophan ratio, and elevated serum inflammatory cytokines. Our findings suggest that these factors correlate with and may predict disease severity. Hyperproduction of pro-inflammatory cytokines such as IL-1β, IL-6, IL-12, IFN-**γ** and TNF-α have been linked to the pathogenesis of tissue injury observed in SARS-CoV-2 induced pneumonia seen in humans[85, 86]. The exact role that these cytokines play in disease pathology is not fully understood therefore expanded cohort sizes of PTM that include both males and females as well as aged animals may reveal answers not only to this question but may also uncover additional clinical manifestations.

Viral dynamics were similar in PTM as we have reported in RhM[14, 15]. Viral RNA, including subgenomic RNA, was consistently detected throughout the first several days of infection. We detected persistent viral titers at multiple sites in some of the animals throughout the course of the study. These data confirm PTM as a robust model of viral infection and replication, similar to RhM, and suggest this model may be used to study novel virus host relationships.

COVID-19 disease is commonly characterized by pulmonary infiltration of inflammatory immune cells[41]. Innate cells, particularly monocytes, are considered important mediators of disease progression[42]. Although infiltrating monocytes were identified in our PTM, T cells were a more dominant cellular infiltrate into lungs as detected in bronchoalveolar lavage sampling. Specifically, we identified a unique population of CD4+/CD8+ double positive T cells (DP T cells) that upregulated inflammatory cytokines such as TNF-⍺ as well as Granzyme B over the course of infection. DP T cells with cytotoxic capacity have been identified in human and animal studies of influenza[87, 88], human studies of HIV[89, 90], as well as animal studies of tuberculosis[48] and SHIV[91]. Traditionally, these cells would be predicted to be major histocompatibility complex class II (MHC-II) restricted CD4 T cells that upregulate CD8 upon activation. Pulmonary infiltrating CD4 T cells with cytotoxic capacity, as measured by Granzyme B, identified in our PTM and recently in humans with severe COVID-19 [25], are a unique and possibly understudied aspect of the disease.

We also identified relatively high magnitude CD4 T cell responses against the virus that localized to the lung 21 days after viral infection. CD8 T cells against the virus were also noted, but at lower frequencies. Many studies have reported antiviral T cells in SARS-CoV-2 convalescent humans, with a relative predominance of CD4 T cells, however these responses are nearly always noted in blood[49, 50]. Thus, our data provide a valuable addition to the data from humans and may suggest important roles for antiviral CD4 T cells in pulmonary sites.

RhM have proved invaluable for testing vaccines and therapeutics[19] due to the robust viral replication and mild but consistent disease seen in this species. However, RhM and other NHP species tested to date do not recapitulate the most severe form of the disease. Based on disease severity with other viruses, we proposed that PTM may provide such a model. Here, we found that PTM largely recapitulate the level of disease severity found in RhM. However, we also found that PTM do demonstrate some important aspects of disease, including the pulmonary infiltration of specific immune cells that may be important in COVID-19 disease. Thus, this species may be complementary to the RhM model for vaccine testing but may also prove uniquely useful for testing certain immune modulating therapeutics.

Taken together, our data define a new animal model for COVID-19. PTM show robust viral replication, SARS-CoV-2 associated pneumonia, and complex innate and adaptive immune responses that may shed light on mechanisms of COVID-19 disease. This model may prove valuable for testing novel immunomodulatory therapeutics and vaccines, including those that modulate pulmonary infiltration of T cells and other inflammatory cells. Finally, our data confirmed COVID-19 associated inflammation was not always resolved 21-dpi, despite no evidence of continued viral replication at that time point. Thus, this model may also be valuable for the study of long-term chronic effects associated with SARS-CoV-2 infection.

## Supporting information

S1 Fig Body temperature and oxygen saturation in SARS-CoV-2 infected pigtail macaques. A. Body temperature measurements before and 1-, 4-, 6-, 14-, and 21-days post (dpi) SARS-CoV-2 infection. B. Saturation of peripheral oxygen (SpO_2_) levels before and 1-, 4-, 6-, 14-, and 21-dpi. Baseline (Day of infection): n = 4, Day 4: n = 4, Day 6: n = 4, Day 14: n = 2, Day 21: n = 2
(TIF)

S2 Fig Radiographs of pigtail macaques (PTM) challenged with SARS-CoV-2. MA27 baseline (A) and 6-days post infection (dpi) (B). MA30 at baseline (C) and 6-dpi (D). MA24 at baseline (E), 6-dpi (F), 14-dpi (G) and 21-dpi (H). MA28 at baseline (I), 6-dpi (J), 14-dpi (K) and 21-dpi (L). Baseline for all four PTM was established 3-days prior to infection.
(TIF)

S3 Fig Gross pathological pulmonary pathology in SARS-CoV-2 infected pigtail macaques (PTM). A-D. Gross pulmonary pathology at 6- (A and B) and 21-days post infection (dpi, C and D). A. MA27, the left caudal lung lobe has multifocal tan-plum areas of consolidation (arrows). Inset: the consolidation extends to the diaphragmatic and medial surface of the left caudal lung. There is no evidence of gross pathology in MA30 (B) or MA24 (C). D. MA28, the laterodorsal aspect of the left caudal lobe contains two small, flat tan foci (arrows). Inset: closer view of tan foci.
(TIF)

S4 Fig Changes in IDO activity post SARS-CoV-2 Infection. (A) Tryptophan (Trp) and (B) Kynurenine (Kyn) levels in plasma before and after SARS-CoV-2 infection. Day 0 = day of infection, Baseline: n = 4, Day 7: n = 4, Day 14: n = 2, Day 21: n = 2. dpi = days post infection.
(TIF)

S5 Fig SARS-CoV-2 peptide specific T cell response in the blood 21-days post infection. Two animals shown (MA24 and MA28 euthanized at 21-dpi). Flow cytometry dot plots showing CD4+ (A) and CD8+ (B) T cell Interferon-**γ** (IFN-*γ*) response to overnight SARS-CoV-2 peptide (spike, membrane, nucleocapsid and envelope) stimulation. No stim o/n = cells incubated overnight without stimulation. Heatmap represents arcsin transformed MFI values.
(TIF)

S6 Fig Gating Strategies. **A**. Example gating of Monocytes: Time (SSC-A vs. Time), Single cells (FSC-H vs. FSC-A), Live cells (FSC-H vs live/dead), Leukocytes (SSC-A vs. FSC-A), CD45+ Leukocytes (SSC-A vs. CD45), HLA-DR^+^ (SSC-A vs. HLA-DR), Monocytes (CD14 vs. CD16): Classical Monocytes: CD14+ CD16-, Intermediate Monocytes: CD14^+^ CD16^+^, Non-classical Monocytes: CD14-CD16^+^. **B.** Example gating of T lymphocytes and Natural killer (NK) cells: Time (SSC-A vs. Time), Single cells (FSC-H vs. FSC-A), Live cells (FSC-H vs. live/dead), Lymphocytes (SSC-A vs. FSC-A), CD45^+^ Lymphocytes (SSC-A vs. CD45), CD3^+/-^ (SSC-A vs. CD3), CD3^+^ T cells (CD8 vs. CD4) T helper: CD4^+^CD8^-^, Cytotoxic T: CD8^+^CD4^-^, Double Positive (DP): CD4^+^CD8^+^, Double negative (DN): CD4^-^CD8^-^, NK cells: CD3^-^HLA-DR^-/low^CD8^+^ (HLA-DR vs. CD8). **C.** Example gating of PD-1^+^ and Ki-67^+^ T cell subsets.
(TIF)

S1 Table Clinical, blood and necropsy observations in pigtail macaques (PTM) challenged with SARS-CoV-2 (n = 4). Values in parenthesis represent days post SARS-CoV-2 infection. Neutrophilia, eosinophilia, basophilia, monocytosis defined as ≥ 2-fold increase over baseline^1^. Lymphocytopenia, monocytopenia, eosinopenia, basopenia defined as a 35% reduction from baseline^1^. CRP: C-reactive protein.
(XLSX)

S2 Table Monocyte Flow Cytometry Panel. (XLSX)

S3 Table Phenotype Flow Cytometry Panel. (XLSX)

S4 Table T cell Flow Cytometry Panel. (XLSX)

S5 Table T cell SARS-CoV-2 Peptide, PMA/Ionomycin Stimulation. (XLSX)

S6 Table Immunohistochemistry Reagent Panel. (XLSX)
