# Supplementary figures and images for: The pigtail macaque (Macaca nemestrina) model of COVID-19 reproduces diverse clinical outcomes and reveals new and complex signatures of disease
Source: PLoS Pathog. 2021 Dec 20;17(12):e1010162. doi: 10.1371/journal.ppat.1010162 (PMC8722729; doi:10.1371/journal.ppat.1010162)

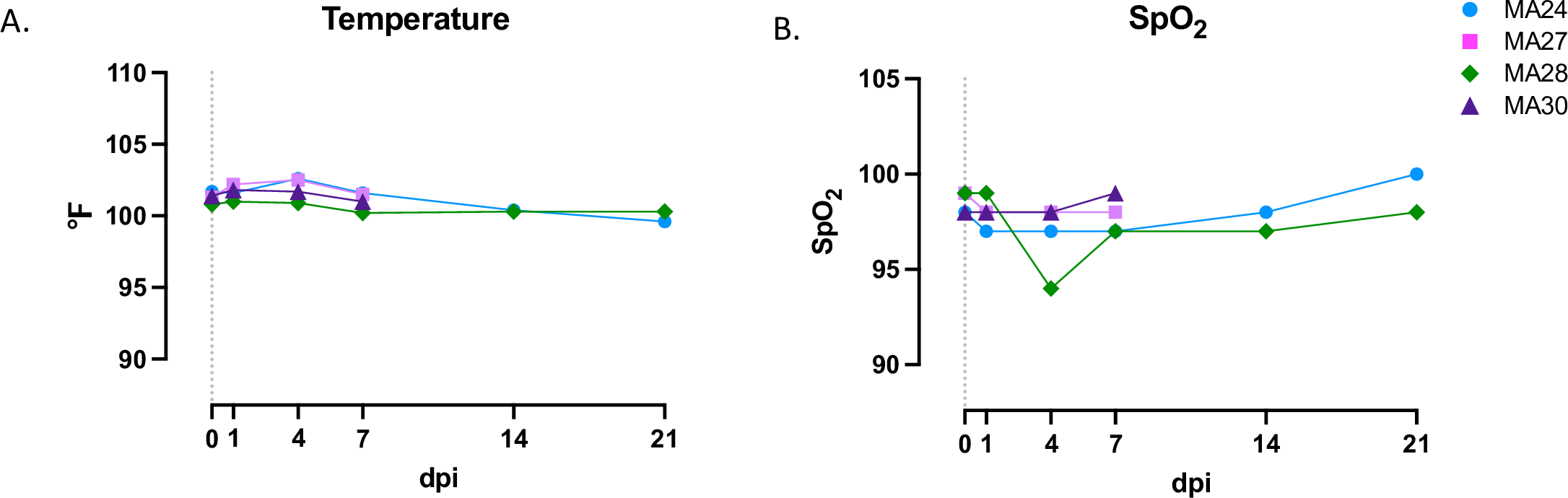

Supplement: S1 Fig — A. Body temperature measurements before and 1-, 4-, 6-, 14-, and 21-days post (dpi) SARS-CoV-2 infection. B. Saturation of peripheral oxygen (SpO2) levels before and 1-, 4-, 6-, 14-, and 21-dpi. Baseline (Day of infection): n = 4, Day 4: n = 4, Day 6: n = 4, Day 14: n = 2, Day 21: n = 2 (TIF) [file ppat.1010162.s001.tif]

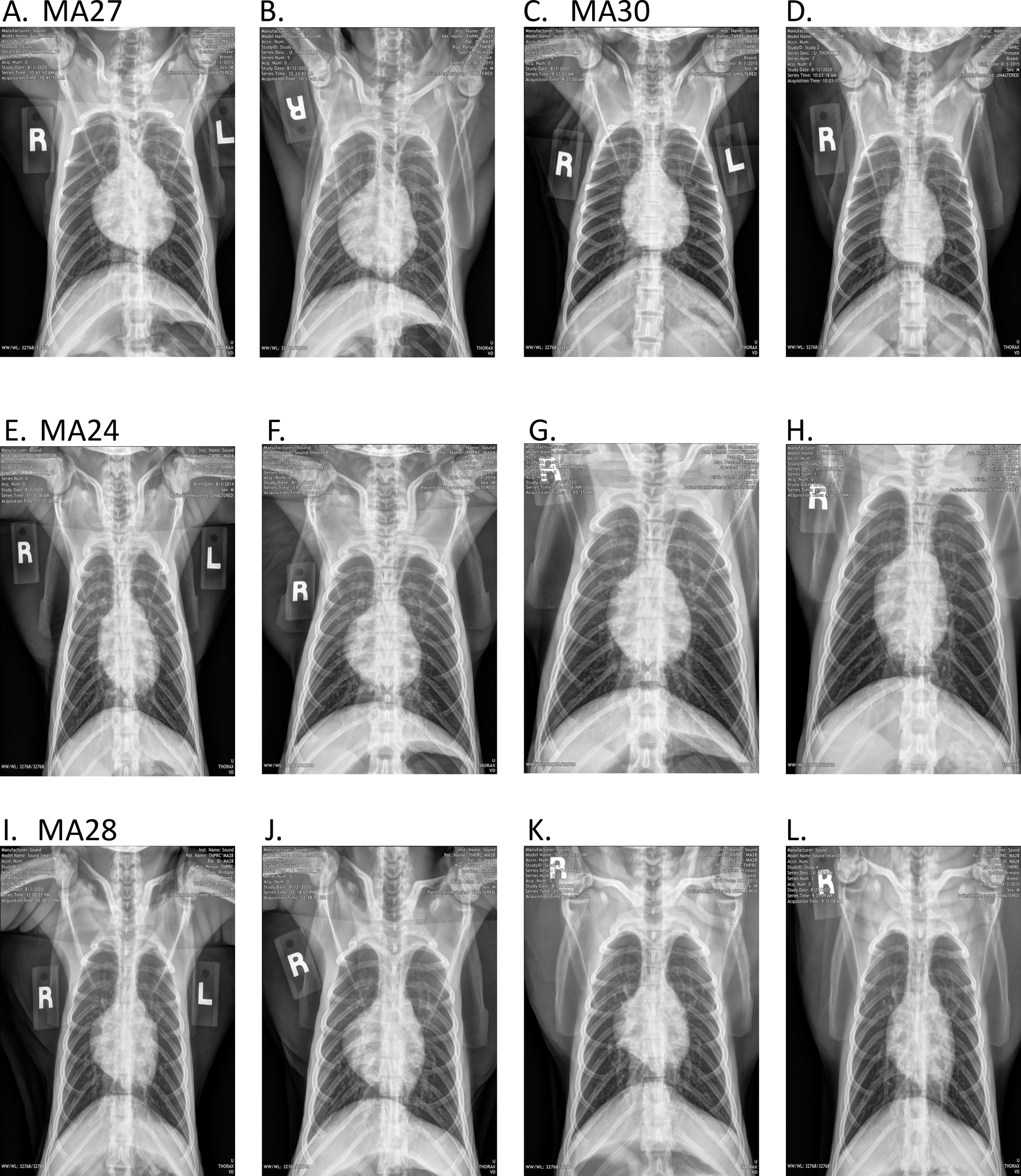

Supplement: S2 Fig — MA27 baseline (A) and 6-days post infection (dpi) (B). MA30 at baseline (C) and 6-dpi (D). MA24 at baseline (E), 6-dpi (F), 14-dpi (G) and 21-dpi (H). MA28 at baseline (I), 6-dpi (J), 14-dpi (K) and 21-dpi (L). Baseline for all four PTM was established 3-days prior to infection. (TIF) [file ppat.1010162.s002.tif]

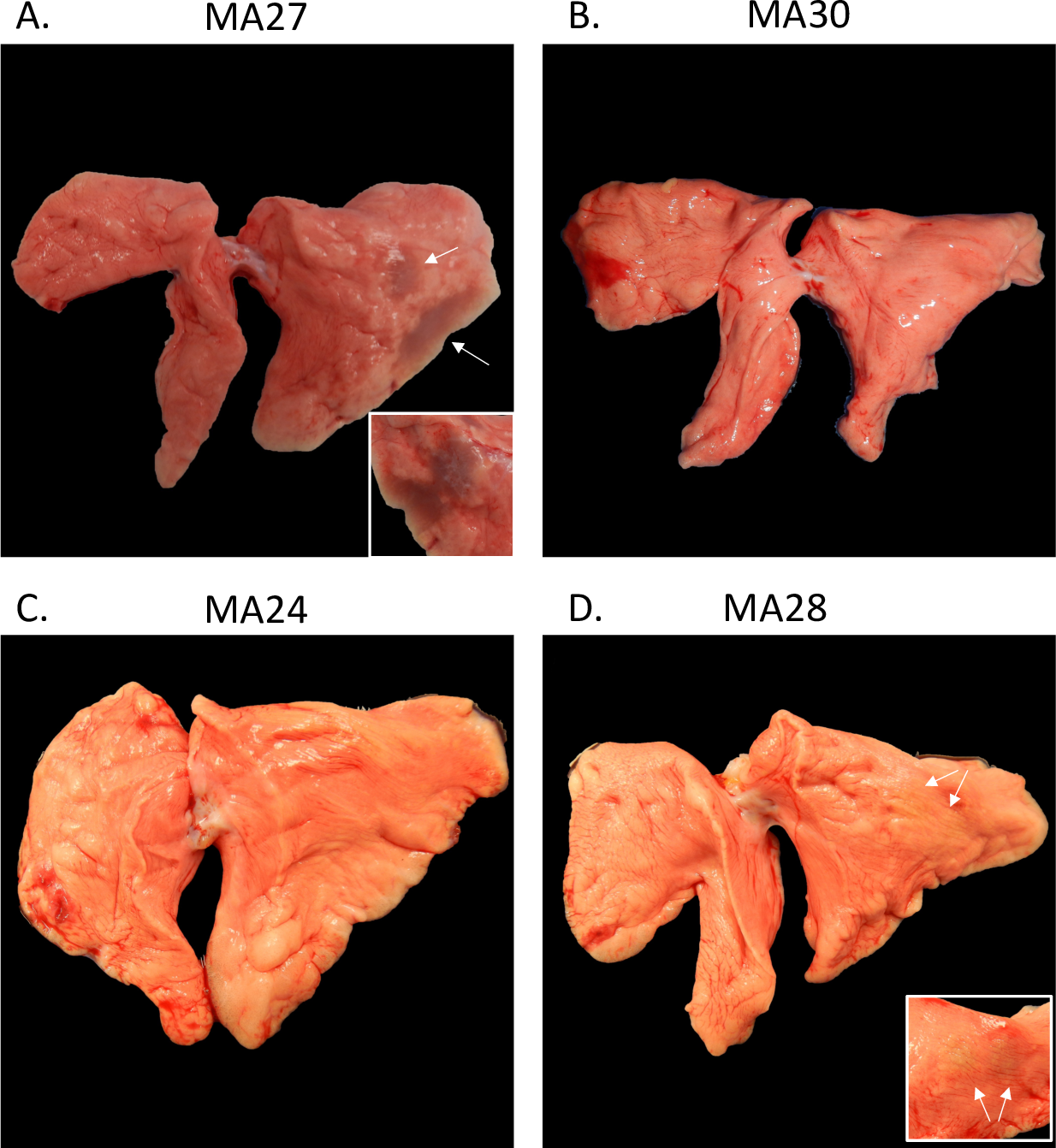

Supplement: S3 Fig — A-D. Gross pulmonary pathology at 6- (A and B) and 21-days post infection (dpi, C and D). A. MA27, the left caudal lung lobe has multifocal tan-plum areas of consolidation (arrows). Inset: the consolidation extends to the diaphragmatic and medial surface of the left caudal lung. There is no evidence of gross pathology in MA30 (B) or MA24 (C). D. MA28, the laterodorsal aspect of the left caudal lobe contains two small, flat tan foci (arrows). Inset: closer view of tan foci. (TIF) [file ppat.1010162.s003.tif]

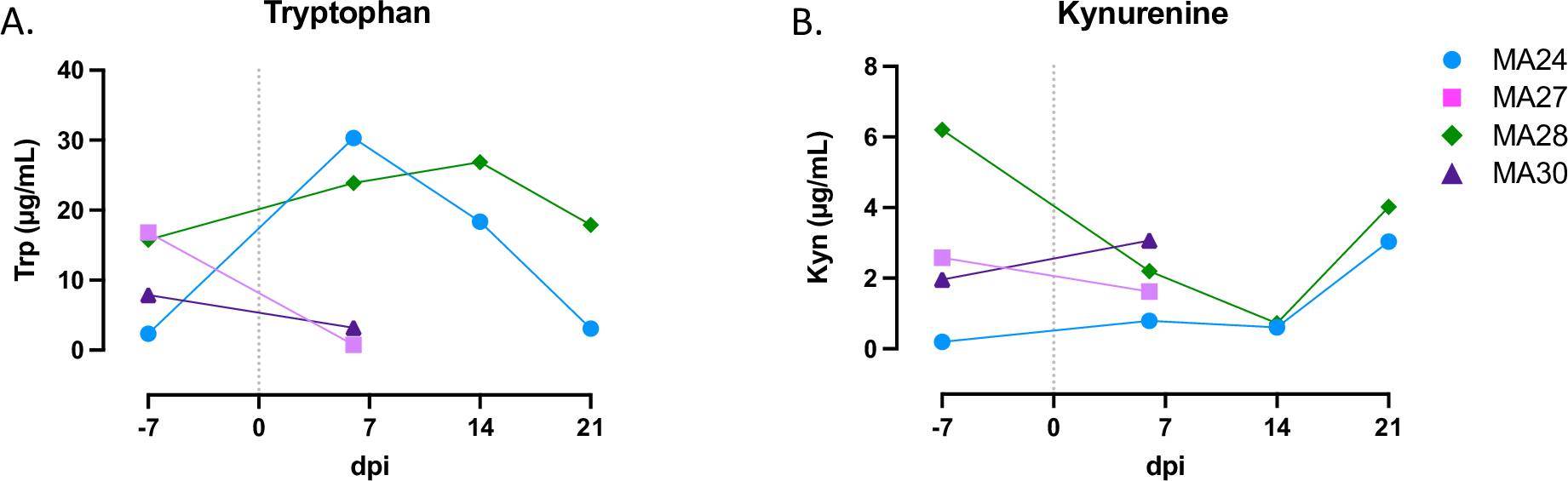

Supplement: S4 Fig — (A) Tryptophan (Trp) and (B) Kynurenine (Kyn) levels in plasma before and after SARS-CoV-2 infection. Day 0 = day of infection, Baseline: n = 4, Day 7: n = 4, Day 14: n = 2, Day 21: n = 2. dpi = days post infection. (TIF) [file ppat.1010162.s004.tif]

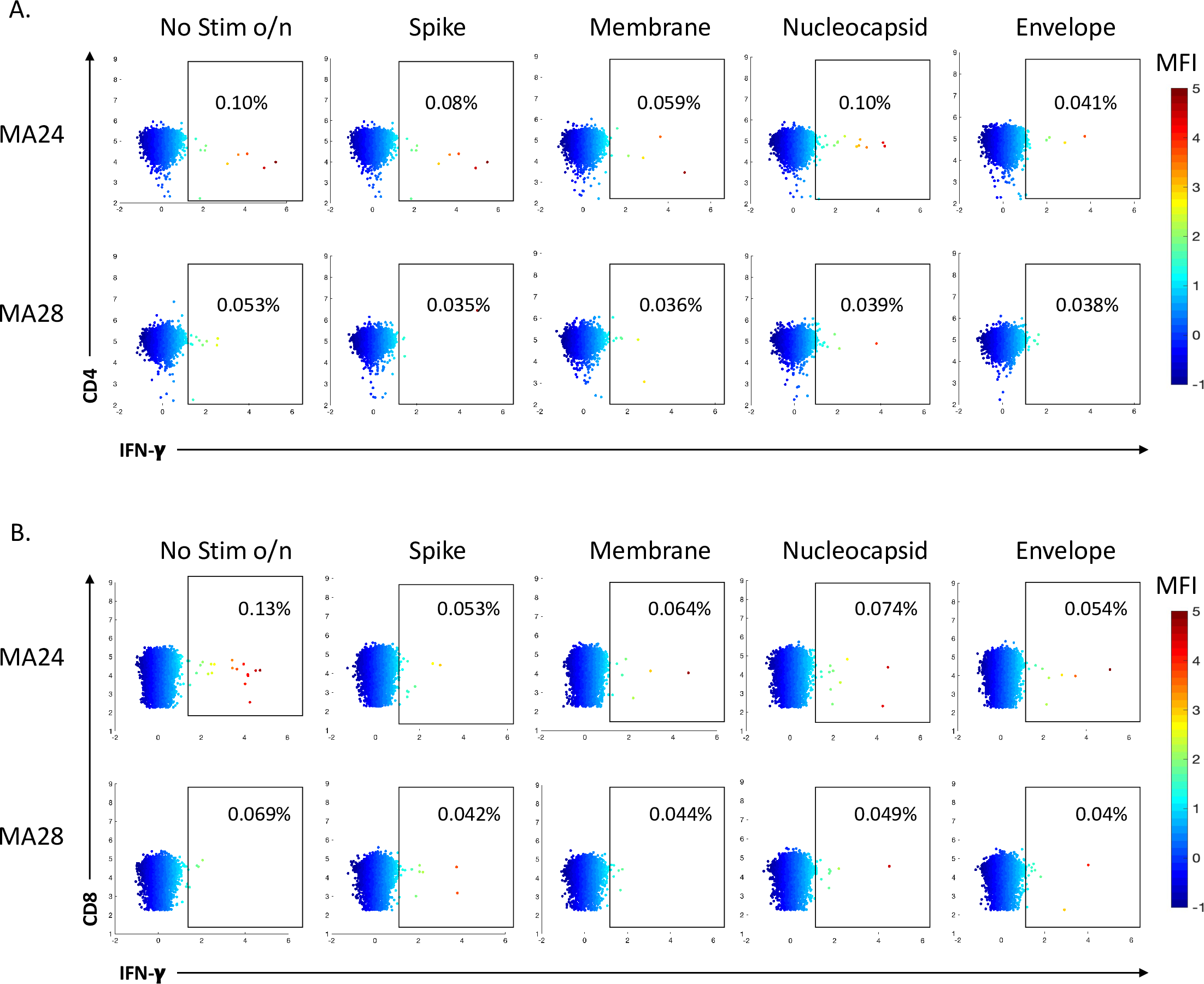

Supplement: S5 Fig — Two animals shown (MA24 and MA28 euthanized at 21-dpi). Flow cytometry dot plots showing CD4+ (A) and CD8+ (B) T cell Interferon-γ (IFN-γ) response to overnight SARS-CoV-2 peptide (spike, membrane, nucleocapsid and envelope) stimulation. No stim o/n = cells incubated overnight without stimulation. Heatmap represents arcsin transformed MFI values. (TIF) [file ppat.1010162.s005.tif]

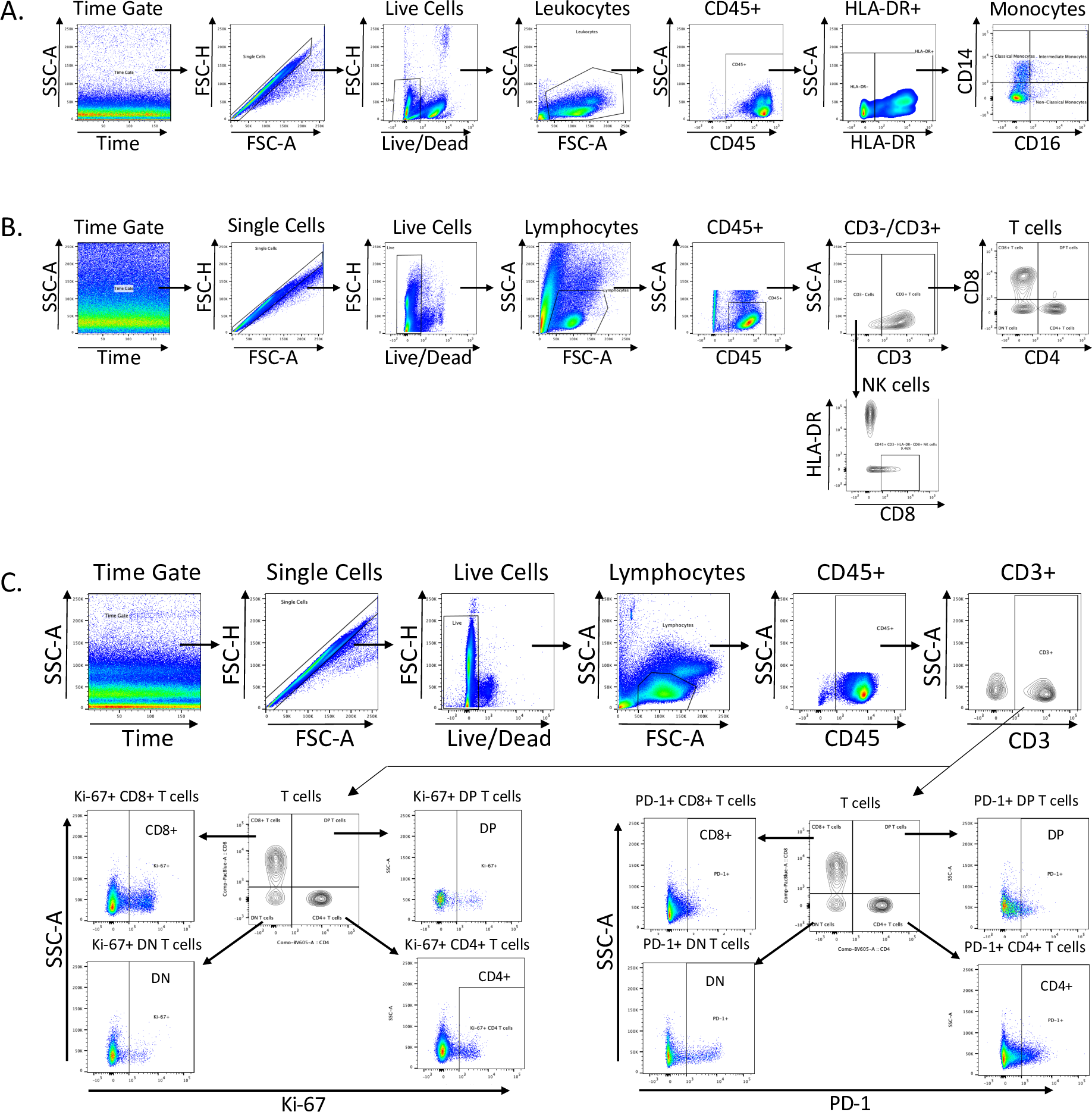

Supplement: S6 Fig — A. Example gating of Monocytes: Time (SSC-A vs. Time), Single cells (FSC-H vs. FSC-A), Live cells (FSC-H vs live/dead), Leukocytes (SSC-A vs. FSC-A), CD45+ Leukocytes (SSC-A vs. CD45), HLA-DR+ (SSC-A vs. HLA-DR), Monocytes (CD14 vs. CD16): Classical Monocytes: CD14+ CD16-, Intermediate Monocytes: CD14+ CD16+, Non-classical Monocytes: CD14-CD16+. B. Example gating of T lymphocytes and Natural killer (NK) cells: Time (SSC-A vs. Time), Single cells (FSC-H vs. FSC-A), Live cells (FSC-H vs. live/dead), Lymphocytes (SSC-A vs. FSC-A), CD45+ Lymphocytes (SSC-A vs. CD45), CD3+/- (SSC-A vs. CD3), CD3+ T cells (CD8 vs. CD4) T helper: CD4+CD8-, Cytotoxic T: CD8+CD4-, Double Positive (DP): CD4+CD8+, Double negative (DN): CD4-CD8-, NK cells: CD3-HLA-DR-/lowCD8+ (HLA-DR vs. CD8). C. Example gating of PD-1+ and Ki-67+ T cell subsets. (TIF) [file ppat.1010162.s006.tif]
